# Identification of Recurrent Mutations in the microRNA-Binding Sites of B-Cell Lymphoma-Associated Genes in Follicular Lymphoma

**DOI:** 10.3390/ijms21228795

**Published:** 2020-11-20

**Authors:** Erika Larrea, Marta Fernandez-Mercado, José Afonso Guerra-Assunção, Jun Wang, Ibai Goicoechea, Ayman Gaafar, Izaskun Ceberio, Carmen Lobo, Jessica Okosun, Anton J. Enright, Jude Fitzgibbon, Charles H. Lawrie

**Affiliations:** 1 Molecular Oncology Group, Biodonostia Research Institute, 20014 San Sebastián, Spain; erika.larrea@gmail.com (E.L.); marfermer@yahoo.es (M.F.-M.); ibai.goicoechea@gmail.com (I.G.); 2 Chinese Institute for Brain Research (CIBR), Beijing 102206, China; 3 School of Life Sciences, Tsinghua University, Beijing 100084, China; 4 Biomedical Engineering, School of Engineering, University of Navarra, 20014 San Sebastian, Spain; 5 Great Ormond Street Institute of Child Health, University College London (UCL), London WC1N 1EH, UK; a.guerra@ucl.ac.uk; 6 Barts Cancer Institute, Queen Mary University of London, London EC1M 6BE, UK; j.a.wang@qmul.ac.uk (J.W.); j.okosun@qmul.ac.uk (J.O.); j.fitzgibbon@qmul.ac.uk (J.F.); 7 Multiple Myeloma Group, Centro de Investigación Médica Aplicada (CIMA), Pamplona, 31008 Navarra, Spain; 8 Department of Pathology, Cruces Hospital, 48903 Bilbao, Spain; ayman.gaafareleraky@osakidetza.eus; 9 Hematology Department, Hospital Universitario Donostia, 20014 San Sebastián, Spain; izaskun.zeberioetxetxipia@osakidetza.eus; 10 Department of Pathology, Hospital Universitario Donostia, 20014 San Sebastián, Spain; mariacarmen.lobomoran@osakidetza.eus; 11 Department of Pathology, University of Cambridge, Cambridge CB2 1QP, UK; aje39@cam.ac.uk; 12 IKERBASQUE, Basque Foundation for Science, 48009 Bilbao, Spain; 13 Radcliffe Department of Medicine, University of Oxford, Oxford OX4 3DU, UK

**Keywords:** follicular lymphoma (FL), diffuse large B-cell lymphoma (DLBCL), microRNA, mutation

## Abstract

Follicular lymphoma (FL) is a common indolent B-cell lymphoma that can transform into the more aggressive transformed FL (tFL). However, the molecular process driving this transformation is uncertain. In this work, we aimed to identify microRNA (miRNA)-binding sites recurrently mutated in follicular lymphoma patients, as well as in transformed FL patients. Using whole-genome sequencing data from FL tumors, we discovered 544 mutations located in bioinformatically predicted microRNA-binding sites. We then studied these specific regions using targeted sequencing in a cohort of 55 FL patients, found 16 recurrent mutations, and identified a further 69 variants. After filtering for QC, we identified 21 genes with mutated miRNA-binding sites that were also enriched for B-cell-associated genes by Gene Ontology. Over 40% of mutations identified in these genes were present exclusively in tFL patients. We validated the predicted miRNA-binding sites of five of the genes by luciferase assay and demonstrated that the identified mutations in *BCL2* and *EZH2* genes impaired the binding efficiency of *miR-5008* and *miR-144* and regulated the endogenous levels of messenger RNA (mRNA).

## 1. Introduction

Follicular lymphoma (FL) is the most common form of low-grade B-cell lymphoma accounting for approximately 20% of all diagnosed lymphomas [1]. Due to its slow progressive nature, FL is often considered an incurable malignancy, with a median life expectancy of approximately 15–20 years [2,3]. However, 5–10% of FL patients undergo high-grade histological transformation into a much more aggressive form of lymphoma with a poorer outcome, generally termed transformed FL (tFL) [4,5,6]. Despite the high frequency of this event, the transformation process is only poorly understood at the molecular level, and no predictive biomarkers currently exist for patients at risk from this phenomenon.

The dysfunctional expression of microRNAs (miRNAs) in B-cell lymphoma is now well established [7]. Our group and others have shown that the pattern of dysfunctional miRNAs in FL differs from that of other B-cell lymphomas including diffuse large B-cell lymphoma (DLBCL) and Burkitt’s lymphoma, as well as between transformed and nontransformed FL cases [8,9,10,11,12,13,14]. Indeed, several studies have proposed a major functional role for miRNAs in the transformation process [8,14]. In addition to changes in the expression of miRNAs in FL, recurrent mutations in the mature sequence of miRNAs have also been associated with FL (and DLBCL) cases [15]. However, the presence of mutations in the miRNA-binding sites of target genes have not yet been explored in FL or indeed any lymphoma.

To address this question, we used whole-genome sequencing (WGS) data to investigate the presence of somatic mutations in miRNA-binding sites from matched FL tissue obtained before and after transformation. Functional testing showed that mutations in *BCL2* and *EZH2*, key genes for lymphomagenesis, impair the binding efficiency of *miR-5008* and *miR-144* and the regulation of endogenous messenger RNA (mRNA) expression.

## 2. Results

### 2.1. Identification of Somatic Mutations in miRNA-Binding Sites of FL

We used whole-genome sequencing data from tumor samples from six FL patients who underwent transformation to high-grade disease to identify somatic mutations located in microRNA binding sites. This discovery cohort consisted on paired samples before and after transformation. From the 2056 somatic mutations identified, approximately half of them were located in coding regions of the genes (47%), whereas 38% of mutations were located in 3′ untranslated regions (UTRs) and 15% were located in 5′ UTRs [16].

Using a bespoke bioinformatic pipeline, we identified 544 mutations (Table S1, Supplementary Materials) located in predicted miRNA-binding sites of 490 different genes (26.5% of total mutations). Nearly all these mutations (92%) were located in 3′ UTRs. Mutations in coding regions of these 490 genes appeared to be mutually exclusive, with only 5% of genes (*n* = 27) having mutations in both coding and 3′ UTR regions. Interestingly, from all the somatic mutations located in 3′ UTR sequences (*n* = 788), 70% arose in predicted miRNA-binding sites (Figure S1, Supplementary Materials). Ontology analysis of the miRNA-binding site mutated genes showed a significant enrichment for pathways of hematological disease (*p* = 2.18 × 10^−4^) and B-cell receptor pathways (*p* = 1.16 × 10^−3^) (Figure S2 and Table S1, Supplementary Materials). Consequently, we designed a panel for targeted sequencing to assess the frequency of the miRNA-binding site mutations in a larger cohort of FL cases.

### 2.2. Genes Showing Recurrent Mutations in miRNA-Binding Sites Are Highly Enriched for Germinal-Center-Like B-Cell Lymphoma Genes

**Figure 1:**
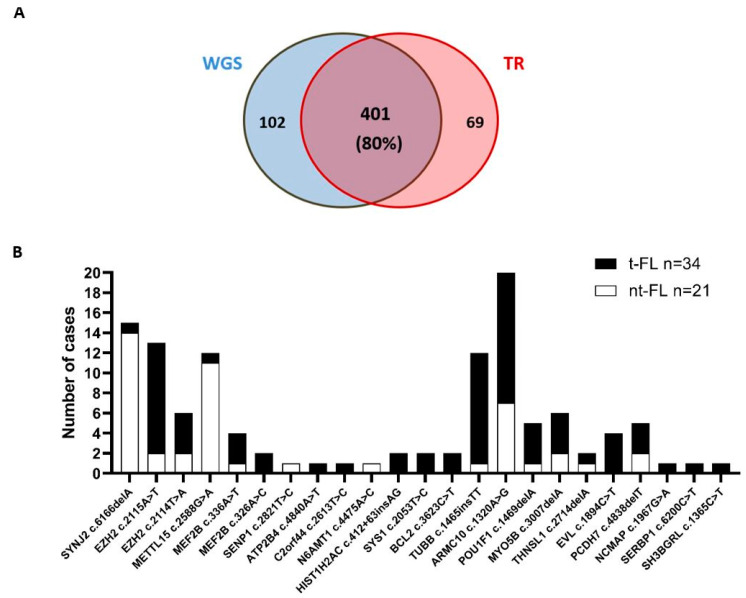
Targeted sequencing results: (**A**) comparison of variants detected by whole-genome sequencing (WGS) and targeted resequencing (TR); (**B**) number of nontransformed (ntFL) and transformed FL cases (tFL) harboring any of the 23 identified recurrent variants after filtering in microRNA (miRNA)-binding sites.

A custom sequencing panel covering >90% of the identified mutations (503/544) was used to sequence a cohort of 55 FL patient samples together with 34 healthy control samples pooled in five samples. From these 55 patients, 34 underwent a histological transformation (tFL) and 21 were classified as non-transforming cases (ntFL). Across all the samples, the mean depth of coverage was 2270× (Figure S3, Supplementary Materials). Eighty percent of the mutations identified by WGS were also identified and validated by targeted sequencing in the original discovery cohort (401/503) (Figure 1A).

From the 503 mutations analyzed, 16 were identified recurrently in validation cohort samples (Table 1). In addition, to the originally identified mutations, a further 69 variants were identified by targeted next-generation sequencing (NGS) as being within predicted miRNA-binding sites. Forty-six of these variants (67%) were identified in more than one sample (Table S2, Supplementary Materials). From the total of 85 identified variants (i.e., 16 recurrent discovery and 69 new validation variants), 36 were removed from further analysis as they were also identified in the pooled healthy control samples (*n* = 34 pooled in five samples). A further 26 were removed as they were present in homopolymer or repetitive regions, which are frequently artefactual [17,18]. The remaining 3 variants consisting of seven of the original identified mutations and 16 of the newly discovered variants were located in 21 different genes (Table S1, Supplementary Materials). Ontology analysis of these 21 genes demonstrated that they were highly enriched for germinal-center-like (GC-like) B-cell lymphoma genes (*p* = 4.39 × 10^−5^). We observed that these variants occurred more commonly in tFL (*n* = 73 variants in 34 cases) compared to non-tFL cases (*n* = 46 variants in 21 cases), and that 10 out of the 23 variants (43%) occurred exclusively in tFL cases (Figure 1B).

### 2.3. In Vitro Luciferase Assays Demonstrated That Mutations in miRNA Binding Sites Interfere with the miRNA Activity

We selected five candidate genes from the 21 genes for further analysis. These genes were selected on the basis that they had mutations in multiple samples (*n* = 11), which were not previously identified as a single-nucleotide polymorphism in the dbSNP database (*n* = 7) [19] (Table S1, Supplementary Materials) and which we could confirm by Sanger sequencing (*n* = 5) (Table 2).

**Figure 2:**
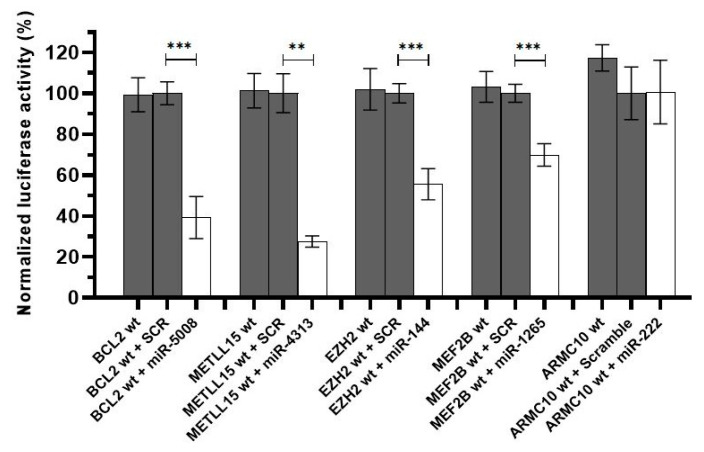
Luciferase results for the wildtype target sequences. Luciferase data showed that the binding sites in *BCL2*, *METTL15*, *EZH2* and *MEF2B* for *miR-5008*, *miR-431*, *miR-144*, and *miR-1265*, respectively, were functional. A downregulation in the luciferase activity can be appreciated under the effect of the miRNA compared to the control conditions. This was not the case for the predicted binding site in *ARMC10* gene. SCR refers to a scramble miRNA sequence used as a negative control. *** *p* < 0.0001, ** *p* < 0.01.

Next, we tested whether or not the predicted miRNA-binding sites where mutations were found were actually functional using a luciferase assay system. We cloned the wildtype sequence of the microRNA-binding sites, flanked by 150 bp upstream and downstream, into the psiCHECK dual-luciferase vector (Figure S4, Supplementary Materials). These constructs were co-transfected into HeK293 cells along with the respective miRNA or a control scramble sequence. The sequences of *BCL2*, *METTL15*, *EZH2*, and *MEF2B* displayed a significant reduction in luciferase output in the presence of their respective cognate miRNA when compared to a scramble control sequence (Figure 2). There was no significant reduction in the *ARMC10/miR-222* combination suggesting that this predicted miRNA-binding site is nonfunctional.

**Figure 3:**
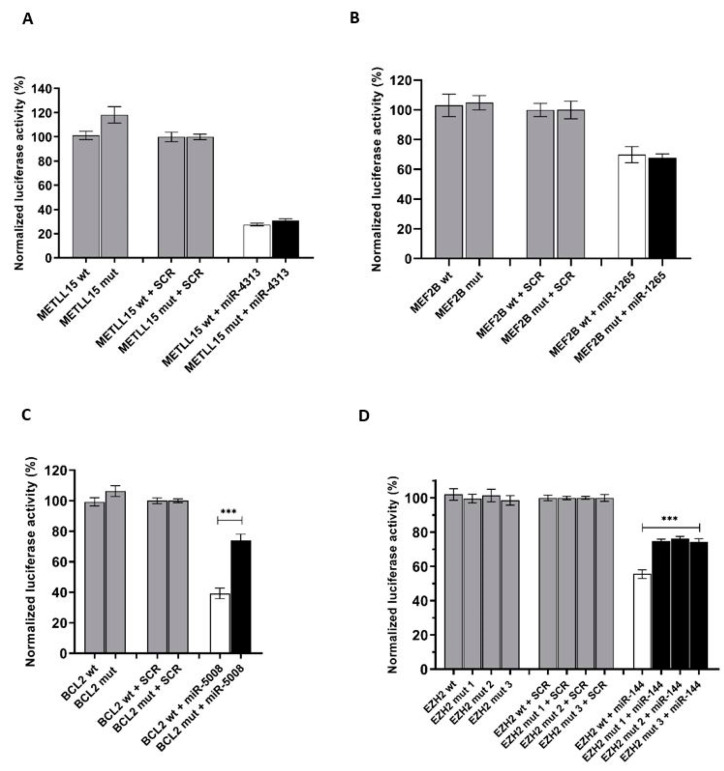
Luciferase results for the mutated target sequences: Luciferase data showed that the mutations identified in *BCL2* (**C**) and *EZH2* (**D**) genes in FL patients negatively interfere with *miR-5008* and *miR-144* binding, respectively, as a significant increase in the luciferase activity could be detected compared to the wildtype condition. In contrast, this was not observed in *METTL15* (**A**) and *MEF2B* (**B**). SCR refers to a scramble miRNA sequence used as a negative control. For *EZH2*, mut 1 refers to c.2115A > T, mut 2 refers to c.2114T > A, and mut 3 refers to c.2114T > C. *** *p* < 0.0001.

Next, we assessed the effect of the identified mutations on the binding affinity of the miRNAs. To do this we compared the luciferase activity between cells transfected with the wild-type target sequence versus cells transfected with the mutated target sequences in the presence of the respective miRNA or scramble sequence. The luciferase output of both *METLL15* and *MEF2B* variants did not significantly change between the mutated sequence and the wild-type sequence (Figure 3).

However, mutations in *BCL2* and *EZH2* genes promoted a significant increase in luciferase activity compared to the wildtype sequence, indicating an impediment in microRNA binding and, subsequently, a less effective regulation of the luciferase gene. We observed this for the c.3623C > T mutation in the *BCL2* gene and c.2115A > T and c.2114T > A mutations in the *EZH2* gene (Figure 3). We additionally tested the c.2114T > C *EZH2* mutation which was previously reported in FL patients [20]. This mutation also resulted in a significant increase in luciferase output when compared to the wildtype sequence.

### 2.4. Testing the Effect of miR-5008 and miR-144 on Basal Target Gene Expression

**Figure 4:**
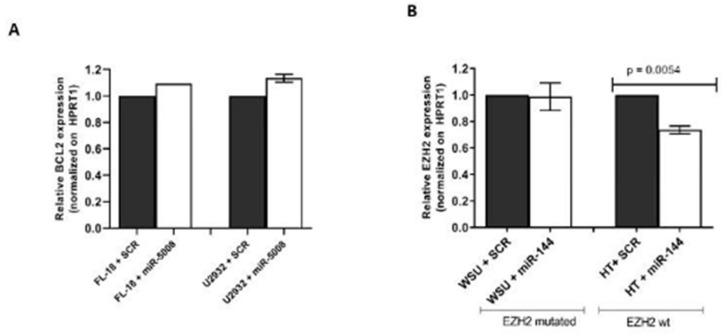
Effect of adding selected miRNAs to endogenous expression levels of target genes. (**A**) *BCL2* expression in lymphoma cells transfected with *miR-5008*. *BCL2* expression levels were measured using RT-qPCR in FL-18 and U2932 lymphoma cell lines. (**B**) *EZH2* expression in lymphoma transfected with miR-144. SCR refers to a scramble miRNA sequence used as a negative control.

In order to test the effect of varying the levels of *miR-5008* on *BCL2* expression, we transfected the lymphoma cell lines FL18 and U2932 with either a mimic of *miR-5008* or a scrambled control sequence. As can be seen from Figure 4A, although there was an increase in *BCL2* expression, this was not significant.

We tested the effect of the addition of *miR-144* to endogenous levels of *EZH2* in the HT (wildtype *EZH2* sequence) and WSU-DLCL2 (mutated *EZH2* sequence) cell lines. As can be seen from Figure 4B, increasing the levels of *miR-144* resulted in a significant decrease in the levels of endogenous *EZH2* mRNA in the HT cell line, which has a wildtype *EZH2*-binding site sequence, but had no effect on the WSU-DLCL2 cell line, which harbors the *c.2115A > T* mutation in the *miR-144*-binding site in *EZH2* gene [21]. This suggests that mutations affecting this binding site promote that *EZH2* is no longer regulated by *miR-144* in lymphoma cells.

## 3. Discussion

It was previously reported that mutations in the binding sites of miRNA-target genes are associated with several cancer types from in silico studies [22,23], as well as experimentally in colorectal cancer [24] and acute myeloid leukaemia (AML) [25]. However, outside of these studies, little has been researched. In this work, we identified mutations in *BCL2* and *EZH2* in FL patients in the *miR-5008-* and *miR-144*-binding sites, respectively, and experimentally demonstrated that these mutations have the potential to impair these miRNAs binding.

*BCL2* and *EZH2* genes are both key genes in the pathogenesis of FL [20,26,27]. The t(14;18) translocation, present in >85% of FL cases, is considered the molecular hallmark of this disease and results in constitutive expression of the antiapoptotic protein BCL2 [28,29,30]. We observed that mutations in the *BCL2* 3´ UTR binding site for *miR-5008* decreased the downregulation of this gene, thereby increasing *BCL2* expression in luciferase assays, although we were not able to confirm this phenomenon in FL18 or U2932 cells, probably due to high basal levels of *BCL2* expression in these t(14;18) cell lines. As the vast majority of FL cases also contain the t(14;18) translocation, the regulatory relevance of this mutation remains speculative. However, it remains possible that this mechanism could be relevant in t(14;18) negative FL, as the mechanism for BCL2 overexpression in these cases currently remains unresolved [31]. Interestingly, the two FL cases that we identified as containing the *c.3623C > T(BCL2)* mutation both underwent high-grade transformation, consistent with the finding that *BCL2* mutations have been reported to have an increased risk increased of transformation [32].

EZH2 is a well-characterized histone methyltransferase in lymphoma that plays a major role in the pathogenesis of FL, and mutations in this gene have been established as an early event in FL lymphomagenesis with activating somatic mutations in the catalytic SET domain of EZH2 in more than 25% of FL patients [20]. Mutations of the *EZH2* gene increase repression via enhanced H3K27 trimethylation, and this transcriptional profile favors proliferation of lymphoma cells and results in the repression of plasma cell differentiation signatures [20,27,33,34,35]. We identified mutations in the Y641 codon (*c.2115A > T*, Y641F and *c.2114T > A*, Y641N) in our study cohort. Interestingly, these activating mutations have previously been reported to prevent Jak2/β-TrCP-mediated degradation of the protein [36]. On the basis of our findings, we propose an additional novel mechanism for these mutations which is to alter the binding affinity of the miRNA *miR-144*. Indeed, *EZH2* was previously reported as a direct target of *miR-144* in cancer [37,38,39]. Some of these studies demonstrated an inverse correlation between *miR-144* and *EZH2* expression in samples of patients, with *miR-144* usually downregulated and *EZH2* upregulated [38,39], as well as a strong correlation with the aggressiveness of the associated tumor [38]. Furthermore, a significant role for *miR-144* in B-cell lymphoma was previously demonstrated [40]. Importantly, *EZH2* expression was found to be significantly higher in FL patients carrying these activating mutations in *EZH2* compared to nonmutated FL patients [41].

In conclusion, in this work, we identified novel recurrent mutations in FL cases that occur in a nonrandom manner in the miRNA-binding sites of genes that are key drivers of FL pathogenesis. This includes the demonstration of a novel regulatory mechanism for arguably two of the most important genes in FL, *BCL2* and *EZH2.* This work suggests that noncoding mutations and specifically mutations in miRNA-binding sites, although often over-looked in genetic studies, are likely to be important factors in lymphomagenesis and cancer in general that surely warrant further investigation.

## 4. Materials and Methods

### 4.1. Patient Selection

A total of 85 retrospectively collected tumor samples (77 frozen and eight formalin-fixed and paraffin-embedded (FFPE)) were obtained from 55 patients diagnosed with FL attending the Barts Cancer Institute, London (*n* = 25), Cruces University Hospital, Bizkaia, Spain (*n* = 4), or Donostia University Hospital, Gipuzkoa, Spain (*n* = 26). Thirty-four of these samples were sampled longitudinally both prior to transformation (i.e., antecedent FL (antFL) and after histological transformation (i.e., tFL). The other 21 FL cases were classified as nontransforming (ntFL) cases on the basis of not having had a recorded transformation event with a minimum follow-up time of 5 years. Pretransformation samples were obtained prior to treatment. Individual data on these patients are given in Table S3 (Supplementary Materials). Written informed consent was obtained from all patients for the inclusion of their samples in this study, and samples were collected in accordance with the Declaration of Helsinki and approved by the local ethics committees (CEIC-Euskadi approval number PI2013038, 2 May2013).

### 4.2. Bioinformatic Identification of miRNA-Binding Site Mutations

Whole-genome sequencing (WGS) data obtained from tumor tissue of six FL patients that underwent transformation and were longitudinally sampled, resulting in 14 samples in total, were generated as previously described [16]. Somatic mutation data from this study were interrogated to identify those located in miRNA-binding sites using a bespoke bioinformatics pipeline. In brief, the mature sequences of all human miRNAs retrieved from miRbase (version 19) [42], along with the genomic coordinates of human UTRs taken from Ensembl bioMart (version 68) [43], were used to produce FASTA files containing UTR sequences without mutations and separate files containing the identified WGS mutations. The TargetScan and miRanda standalone algorithms were then used to predict target sites in the generated sequence files [44]. Only mutations located among the 75% most confident target sites for each miRNA were considered. The miRanda target prediction algorithm was then employed on these targets to assess the thermodynamic stability and the effects of the mutations on potential miRNA binding to the target [45]. Ontology analysis of mutations identified in predicted miRNA-binding sites was carried out using Ingenuity Pathway Analysis tool [46].

### 4.3. Ampliseq (Ion Torrent) Panel NGS

The Ion Ampliseq Designer v3.4 tool was used to design a custom panel containing the mutations identified by WGS (*n* = 544). The panel consisted of a single pool of 482 separate amplicons (125–175 bp in length), covering a total of 57 kb, and included 503 of the 544 (92%) of the originally identified mutations. The panel was used to sequence 85 samples obtained from 55 patients consisting of 21 ntFL cases, 27 paired tFL, and matched antecedent FL samples, one longitudinally sampled tFL case that was sampled at time of diagnosis, 1 year post diagnosis and 2 years post diagnosis and post transformation, antecedent samples from four tFL cases, and two samples from tFL cases without the corresponding antecedent sample. In addition, five pools of DNA obtained from the peripheral blood of 34 healthy controls were sequenced. Samples were sequenced either using an Ion Torrent Personal Genome Machine (PGM) (*n* = 70) or an Ion Proton machine (*n* = 15). Signal processing and base calling were performed within the Torrent Server using default parameters. After demultiplexing, reads were aligned against the hg19 version of the human genome, and variants were identified using the Ion Reporter v5.2 online tool using somatic low stringency parameters from the Torrent Variant Caller 4.4 plugin. Only variants with a coverage >30× and a VAF >5% were considered in this analysis.

### 4.4. Luciferase Assays

Luciferase assays were performed on the predicted miRNA-binding sites of five genes (*ARMC10*, *BCL2*, *METTL15*, *EZH2*, and *MEF2B*) to assess the functional consequences of identified mutations on miRNA-binding affinity. The respective predicted miRNA-binding site sequences were cloned, with and without the identified mutations, along with 150 bp of flanking sequence, immediately downstream of the luciferase reporter gene of the psiCHECK-2 dual-luciferase vector (Promega) (Figure S4, Supplementary Materials).

In brief, approximately 10^5^ HEK293 cells, a kind gift from Prof. Adrian Harris (University of Oxford, Oxford, UK) were transfected with individual psiCHECK-2 constructs (200 ng/mL transfection media) along with 50 nM of either the respective miRNA mimic or a scramble miRNA control sequence (miRIDIAN miRNA mimics, Dharmacon, Lafayette, CO, USA). Transfections were performed in biological triplicate and 48 h post-transfection cells were harvested and lysed with Dual-Luciferase Reporter Assay buffer according to the manufacturer’s protocol (Promega, Madison, WI, USA). Measurements were carried out in triplicate using a PHERAstar microplate reader (BMG LABTECH, Ortenberg, Germany). *Renilla* luciferase activity was normalized to the firefly luminescence measurement for each well. Results were compared using Mann–Whitney U test carried out in GraphPad Prism software (version 5.01).

### 4.5. Cell Culture and Transfection

WSU-DLCL2 is a germinal center (GC)-type DLBCL cell line containing the t(14; 18)(q32; q21) translocation and the Y641F mutation in the *EZH2* gene. The HT cell line is also GC-type DLBCL but contains the wildtype sequence of the *EZH2* gene. Both cell lines were obtained from the Barts Cancer Institute. FL-18 is a follicular lymphoma cell line carrying the t(14;18)(q32;q21) translocation [47], and U2932 is a DLBCL cell line that overexpresses BCL2 [48]. All the cell lines were maintained in RPMI medium with 10% of FBS, 1% l-glutamine, and 1% penicillin–streptomycin (Gibco™, Thermo Fisher Scientific, DE, USA).

Cells were transfected with either 1 µM *hsa-miR-144-3p* (WSU-DLCL2 and HT) or *hsa-miR-5008-5p* (FL-18 and U2932) mimic or a scramble control (miRIDIAN miRNA mimics, Dharmacon). Transfections were carried out in biological triplicate via electroporation using a 4D-Nucleofector machine (Amaxa Biosystems, Cologne, Germany). Cells were harvested 24 h after transfection, and total RNA was extracted using TRIzol reagent (Invitrogen, Carlsbad, CA, USA).

### 4.6. qRT-PCR

Total RNA was used as a template for reverse transcription using the High-Capacity Reverse Transcription Kit (Applied Biosystems, Foster City, CA, USA). PCR was carried out in triplicate using a specific Taqman probe for EZH2 (Hs00544830_m1) and BCL2 (Hs00608023_m1) according to the manufacturer´s protocol (ThermoFisher Scientific, Waltham, MA, USA) on a CFX Connect Real-Time PCR Detection System (Bio-Rad, Hercules, CA, USA). Hypoxanthine phosphoribosyl transferase 1 (*HPRT1*) and TATA-binding protein (*TBP*) were used individually as housekeeping controls, as the Ct values for these genes were similar to basal *EZH2* expression levels. Relative expression was calculated using the 2^−ΔCt^ method using the median value of both housekeeping controls. For the statistical analysis, the paired *t*-test was applied using GraphPad Prism software (version 5.01).

## Figures and Tables

**Table 1:** Mutations from the original discovery cohort recurrently identified in the validation cohort.

Gene	Mutated Position	Mutation	Location	miRNA	Cases (*n* = 55)
*EZH2*	chr7:148508727	T/A	Exon	*hsa-mir-144*	8
*ARMC10*	chr7:102739179	A/G	3′ UTR	*hsa-mir-222*	18
*TUBB*	chr6:30692754	C/CTT	3′ UTR	*hsa-mir-1302*	7
*MEF2B*	chr19:19260045	T/A	Exon	*hsa-mir-1265*	3
*METTL15*	chr11:28353434	G/A	3′ UTR	*hsa-mir-4313*	12
*ZNF195*	chr11:3380000	t/TC	3′ UTR	*hsa-mir-1915*	16
*BCL2*	chr18:60793447	G/A	3′ UTR	*hsa-mir-5008*	2
*THOC3*	chr5:175386586	A/G	3′ UTR	*hsa-mir-371a*	15
*TXNDC2*	chr18:9887493	T/C	Exon	*hsa-mir-2110*	3
*PCDH7*	chr4:30732983	GTA/G	Intron	*hsa-mir-329*	4
*RC3H1*	chr1:173901940	A/AAAT	3′ UTR	*hsa-mir-548an*	2
*AQP3*	chr9:33441702	C/A	3′ UTR	*hsa-mir-146b*	8
*DPY19L2*	chr12:63953768	T/C	3′ UTR	*hsa-mir-1303*	11
*MYO5B*	chr18:47352742	T/G	3′ UTR	*hsa-mir-216b*	55
*MYO5B*	chr18:47352754	A/G	3′ UTR	*hsa-mir-2681*	55
*YY2*	chrX:21876221	A/G	3′ UTR	*hsa-mir-448*	36

The table shows the mutated position in the genome, the mutation identified, the location in the gene, the microRNA targeting this region, and the number of cases identified. UTR, untranslated region.

**Table 2:** Selected candidate genes for functional testing.

Gene	Predicted miRNA Binding Site	miRNA	Mutation	Total Patients (*n* = 55) (%)	tFL/ntFL
*ARMC10*	chr7:102739177–102739198	*hsa-mir-222*	c.1320A > G	18 (33)	13/7
*BCL2*	chr18:60793436–60793458	*hsa-mir-5008*	c.3623C > T	2 (4)	2/0
*METTL15*	chr11:28353429–28353448	*hsa-mir-4313*	c.2588G > A	12 (22)	1/11
*EZH2*	chr7:148508722–148508742	*hsa-mir-144*	c.2115A > T	8 (15)	11/2
*EZH2*	chr7:148508722–148508742	*hsa-mir-144*	c.2114T > A	5 (9)	4/2
*MEF2B*	chr19:19260038–19260055	*hsa-mir-1265*	c.336A > T	3 (6)	3/1

These genes were prioritized from the 21 genes on the basis of their frequency (present in more than one patient), while not being reported as common single-nucleotide polymorphisms (SNPs) in the dbSNP database (153) and able to be validated by Sanger sequencing. Therefore, a total of five genes (*ARMC10*, *BCL2*, *METTL15*, *EZH2*, and *MEF2B*) were tested using the luciferase reporter system.

## Supplementary Materials

The following are available online at https://www.mdpi.com/1422-0067/21/22/8795/s1: Figure S1: Distribution of the frequency of the mutations (*n* = 2056) identified using WGS in 6 FL cases in protein coding genes; Figure S2. B-cell receptor signalling pathway; Figure S3: Sequencing coverage archived using the custom targeted sequencing panel; Figure S4. Schematic diagram of the construct for luciferase assays in psiCHECK-2 vector; Table S1. Mutations identified in predicted miRNA-binding sites in the WGS datatitle; Table S2. Recurrent mutations identified by targeted sequencing in the validation cohort and additional variants within predicted miRNA-binding sitestitle; Table S3. Sample detailed information.
