# Supplementary material for: Identification of Recurrent Mutations in the microRNA-Binding Sites of B-Cell Lymphoma-Associated Genes in Follicular Lymphoma
Source: Int J Mol Sci. 2020 Nov 20;21(22):8795. doi: 10.3390/ijms21228795 (PMC7699894; doi:10.3390/ijms21228795)
Supplement: Supplementary file 1 [file ijms-21-08795-s001.zip › ijms-992088-supplementary.docx]

Supplementary

Identification of Recurrent Mutations in the microRNA-Binding Sites of B-Cell Lymphoma-Associated Genes in Follicular Lymphoma

Erika Larrea ^1,2,3^, Marta Fernandez-Mercado ^1,4^, José Afonso Guerra-Assunção ^5^ Jun Wang ^6^, Ibai Goicoechea ^1,7^, Ayman Gaafar ^8^, Izaskun Ceberio ^9^, Carmen Lobo ^10^, Jessica Okosun ^6^ and Anton J. Enright ^11^, Jude Fitzgibbon ^6^ and Charles H. Lawrie ^1,12,13,^*

^1^  Molecular Oncology Group, Biodonostia Research Institute, 20014 San Sebastián, Spain; [erika.larrea@gmail.com](mailto:erika.larrea@gmail.com) (E.L.); [marfermer@yahoo.es](mailto:marfermer@yahoo.es) (M.F.-M.); ibai.goicoechea@gmail.com (I.G.)

^2^  Chinese Institute for Brain Research (CIBR), Beijing 102206, China

^3^ School of Life Sciences, Tsinghua University, Beijing 100084, China

^4^ Biomedical Engineering, School of Engineering, University of Navarra, 20014 San Sebastian, Spain

^5^ Great Ormond Street Institute of Child Health, University College London (UCL), London WC1N 1EH, UK a.guerra@ucl.ac.uk (J.A.G.-A.)

^6^ Barts Cancer Institute, Queen Mary University of London, London EC1M 6BE UK; [j.a.wang@qmul.ac.uk](mailto:j.a.wang@qmul.ac.uk) (J.W.); [j.okosun@qmul.ac.uk](mailto:j.okosun@qmul.ac.uk) (J.O.); [j.fitzgibbon@qmul.ac.uk](mailto:j.fitzgibbon@qmul.ac.uk) (J.F.)

^7^ Multiple Myeloma Group, Centro de Investigación Médica Aplicada (CIMA), Pamplona, 31008 Navarra, Spain

^8^ Department of Pathology, Cruces Hospital, 48903 Bilbao, Spain; [ayman.gaafareleraky@osakidetza.eus](mailto:ayman.gaafareleraky@osakidetza.eus)

^9^ Hematology Department, Hospital Universitario Donostia, 20014 San Sebastián, Spain; [izaskun.zeberioetxetxipia@osakidetza.eus](mailto:izaskun.zeberioetxetxipia@osakidetza.eus)

^10^ Department of Pathology, Hospital Universitario Donostia, 20014 San Sebastián, Spain; [mariacarmen.lobomoran@osakidetza.eus](mailto:mariacarmen.lobomoran@osakidetza.eus)

^11^ Department of Pathology, University of Cambridge, Cambridge CB2 1QP, UK; [aje39@cam.ac.uk](mailto:aje39@cam.ac.uk)

^12^ IKERBASQUE, Basque Foundation for Science, 48009 Bilbao, Spain

^13^ Radcliffe Department of Medicine, University of Oxford, Oxford OX4 3DU, UK

***** Correspondence: [charles.lawrie@biodonostia.org](mailto:charles.lawrie@biodonostia.org); Tel.: +34-943-006138

Received: 22 October 2020; Accepted: 18 November 2020; Published: date

Figure S1-S4

Tables S1-S3

**Table 1.** Mutations identified in predicted miRNA-binding sites in the WGS data.

| **Chr** | **Position** | **miRNA** | **Reference** | **Mutated** | **Gene** | **Location** | **Sample ID** |
| --- | --- | --- | --- | --- | --- | --- | --- |
| 1 | 160247610 | *hsa-mir-3189* | T | C | *PEX19* | 3utr | S1_FL |
| 1 | 160247627 | *hsa-mir-125b* | T | A | *PEX19* | 3utr | S1_FL |
| 1 | 179072649 | *hsa-mir-4759* | T | G | *ABL2* | 3utr | S3_tFL |
| 1 | 230838449 | *hsa-mir-181a* | C | T | *AGT* | 3utr | S4_FL,S4_tFL |
| 1 | 243665741 | *hsa-mir-1238* | C | G | ***AKT3*** | 3utr | S2_tFL |
| 1 | 16863809 | *hsa-mir-125a* | C | T | *AL355149.1* | 3utr | S4_tFL |
| 1 | 203709122 | *hsa-mir-2682* | A | C | *ATP2B4* | 3utr | S1_tFL |
| 1 | 85732702 | *hsa-mir-495* | A | C | ***BCL10*** | 3utr | S4_FL,S4_tFL |
| 1 | 52553074 | *hsa-mir-148a* | C | T | *BTF3L4* | 3utr | S6_FL |
| 1 | 226790202 | *hsa-mir-30a* | T | C | *C1orf95* | 3utr | S3_tFL |
| 1 | 19665861 | *hsa-mir-4779* | A | G | *CAPZB* | 3utr | S6_FL,S6_tFL |
| 1 | 115126456 | *hsa-mir-548at* | AAC | A | *DENND2C* | 3utr | S3_tFL |
| 1 | 244869281 | *hsa-mir-103a* | T | A | *PPPDE1* | 3utr | S3_FL |
| 1 | 3801141 | *hsa-mir-10a* | T | G | *DFFB* | 3utr | S6_FL,S6_tFL |
| 1 | 1271039 | *hsa-mir-185* | C | A | *DVL1* | 3utr | S3_tFL |
| 1 | 184660899 | *hsa-mir-4279* | T | G | *EDEM3* | 3utr | S6_FL |
| 1 | 201985852 | *hsa-mir-1262* | C | T | ***ELF3*** | 3utr | S2_tFL |
| 1 | 19545113 | *hsa-mir-495* | A | G | *KIAA0090* | 3utr | S2_FL1,S2_FL2,S2_FL3,S2_tFL |
| 1 | 224346009 | *hsa-mir-1277* | A | C | *FBXO28* | 3utr | S4_tFL |
| 1 | 89473256 | *hsa-mir-3148* | A | C | *GBP3* | 3utr | S6_FL |
| 1 | 202098358 | *hsa-mir-3614* | A | G | *GPR37L1* | 3utr | S2_tFL |
| 1 | 236713460 | *hsa-mir-1244* | T | C | *HEATR1* | 3utr | S4_tFL |
| 1 | 149859044 | *hsa-mir-1257* | G | GGTGA | *HIST2H2AB* | 3utr | S6_tFL |
| 1 | 228369247 | *hsa-mir-3140* | C | A | *IBA57* | 3utr | S6_FL,S6_tFL |
| 1 | 206941015 | *hsa-mir-194* | C | T | *IL10* | 3utr | S2_FL1,S2_FL2,S2_FL3,S2_tFL |
| 1 | 209959244 | *hsa-mir-1292* | G | A | *IRF6* | 3utr | S2_FL1,S2_FL2,S2_FL3,S2_tFL |
| 1 | 65299513 | *hsa-mir-148b* | A | C | ***JAK1*** | 3utr | S6_FL |
| 1 | 160009034 | *hsa-mir-1304* | C | A | *KCNJ10* | 3utr | S2_FL1,S2_FL2,S2_FL3,S2_tFL |
| 1 | 233807805 | *hsa-mir-376c* | T | C | *KCNK1* | 3utr | S1_FL,S1_tFL |
| 1 | 67413810 | *hsa-mir-132* | TG | T | *MIER1* | 3utr | S1_tFL |
| 1 | 2522886 | *hsa-mir-1266* | T | G | *C1orf93* | 3utr | S4_FL |
| 1 | 158819031 | *hsa-mir-4684* | A | G | *MNDA* | 3utr | S5_FL,S5_tFL |
| 1 | 11846026 | *hsa-mir-1245a* | G | T | *MTHFR* | 3utr | S1_tFL |
| 1 | 24933789 | *hsa-mir-4781* | G | A | *C1orf130* | 3utr | S2_FL2 |
| 1 | 226548564 | *hsa-mir-1252* | A | C | *PARP1* | 3utr | S1_FL,S1_tFL |
| 1 | 3351616 | *hsa-mir-1249* | G | A | *PRDM16* | 3utr | S3_FL,S3_tFL |
| 1 | 79004088 | *hsa-mir-483* | G | A | *PTGFR* | 3utr | S2_tFL |
| 1 | 89446130 | *hsa-mir-129* | A | T | *RBMXL1* | 3utr | S6_FL |
| 1 | 173901940 | *hsa-mir-548an* | AAAT | A | *RC3H1* | 3utr | S4_FL |
| 1 | 38078213 | *hsa-mir-147a* | G | A | *RSPO1* | 3utr | S3_FL,S3_tFL |
| 1 | 67874055 | *hsa-mir-150* | C | G | *SERBP1* | 3utr | S1_tFL |
| 1 | 93615674 | *hsa-mir-204* | A | G | *TMED5* | 3utr | S5_tFL |
| 1 | 9664937 | *hsa-mir-15a* | A | G | *TMEM201* | 3utr | S5_tFL |
| 1 | 155155030 | *hsa-mir-4475* | C | T | *TRIM46* | 3utr | S6_FL |
| 1 | 247132908 | *hsa-mir-3908* | A | ATTCCACAGT… | *ZNF670* | 3utr | S4_FL |
| 1 | 71530387 | *hsa-mir-145* | T | G | *ZRANB2* | 3utr | S1_FL,S1_tFL |
| 1 | 110038447 | *hsa-mir-127* | C | T | *CYB561D1* | coding | S1_tFL |
| 1 | 171123340 | *hsa-mir-2355* | A | G | *FMO6P* | coding | S2_FL2,S2_FL3 |
| 2 | 9546301 | *hsa-mir-146a* | A | C | *ITGB1BP1* | 3utr | S5_tFL |
| 2 | 9546324 | *hsa-mir-1245b* | GTGTT | G | *ITGB1BP1* | 3utr | S5_tFL |
| 2 | 197705581 | *hsa-mir-758* | T | C | *PGAP1* | 3utr | S4_FL,S4_tFL |
| 2 | 197712238 | *hsa-mir-3134* | T | A | *PGAP1* | 3utr | S5_tFL |
| 2 | 86365110 | *hsa-mir-3152* | C | T | *PTCD3* | 3utr | S2_FL3 |
| 2 | 86365781 | *hsa-mir-2355* | AG | A | *PTCD3* | 3utr | S1_tFL |
| 2 | 85569666 | *hsa-mir-146a* | C | T | *RETSAT* | 3utr | S5_FL |
| 2 | 85570186 | *hsa-mir-1207* | G | A | *RETSAT* | 3utr | S3_FL |
| 2 | 204259279 | *hsa-mir-205* | A | T | *RAPH1* | 3utr | S2_FL2,S2_FL3 |
| 2 | 37430689 | *hsa-mir-135a* | C | A | *AC007390.5* | 3utr | S2_FL2,S2_FL3 |
| 2 | 132021476 | *hsa-mir-942* | C | T | *AC131180.2* | 3utr | S4_tFL |
| 2 | 127805769 | *hsa-mir-1915* | G | A | *BIN1* | 3utr | S5_FL |
| 2 | 203429766 | *hsa-mir-1273f* | C | T | ***BMPR2*** | 3utr | S1_tFL |
| 2 | 24253440 | *hsa-mir-302a* | T | G | *C2orf44* | 3utr | S3_tFL |
| 2 | 188210007 | *hsa-mir-2681* | T | C | *CALCRL* | 3utr | S1_FL,S1_tFL |
| 2 | 219883372 | *hsa-mir-639* | T | A | *CCDC108* | 3utr | S4_tFL |
| 2 | 159027687 | *hsa-mir-1587* | T | A | *CCDC148* | 3utr | S6_FL,S6_tFL |
| 2 | 70523109 | *hsa-mir-3150a* | G | TATCAATTTC… | *FAM136A* | 3utr | S6_FL |
| 2 | 24286356 | *hsa-mir-124* | A | G | *FKBP1B* | 3utr | S3_tFL |
| 2 | 114258286 | *hsa-mir-4263* | TAGAA | T | *FOXD4L1* | 3utr | S4_FL |
| 2 | 70107498 | *hsa-mir-126* | T | C | *GMCL1* | 3utr | S1_tFL |
| 2 | 80525445 | *hsa-mir-3125* | T | G | *LRRTM1* | 3utr | S4_FL,S4_tFL |
| 2 | 20192847 | *hsa-mir-30a* | T | A | *MATN3* | 3utr | S4_FL,S4_tFL |
| 2 | 128605190 | *hsa-mir-1254* | A | T | *POLR2D* | 3utr | S1_FL,S1_tFL |
| 2 | 210885346 | *hsa-mir-3152* | T | G | *RPE* | 3utr | S4_tFL |
| 2 | 10270375 | *hsa-mir-188* | A | G | *RRM2* | 3utr | S1_FL,S1_tFL |
| 2 | 223423656 | *hsa-mir-202* | G | T | *SGPP2* | 3utr | S1_tFL |
| 2 | 139328316 | *hsa-mir-3974* | A | G | *SPOPL* | 3utr | S2_FL3 |
| 2 | 54480995 | *hsa-mir-34a* | A | T | *TSPYL6* | 3utr | S6_tFL |
| 2 | 242814094 | *hsa-mir-4529* | C | A | *C2orf85* | coding | S1_FL,S1_tFL |
| 3 | 58703311 | *hsa-mir-1303* | G | A | *C3orf67* | 3utr | S3_FL,S3_tFL |
| 3 | 58727863 | *hsa-mir-141* | TTGAA | T | *C3orf67* | 3utr | S1_FL,S1_tFL |
| 3 | 58847570 | *hsa-mir-129* | C | G | *C3orf67* | 3utr | S1_tFL |
| 3 | 171574589 | *hsa-mir-142* | C | CTT | *TMEM212* | 3utr | S6_FL |
| 3 | 171574683 | *hsa-mir-4666a* | G | A | *TMEM212* | 3utr | S2_FL1,S2_FL2,S2_FL3,S2_tFL |
| 3 | 119139312 | *hsa-mir-3146* | C | T | *ARHGAP31* | 3utr | S5_FL |
| 3 | 128758938 | *hsa-mir-1205* | T | G | *CCDC48* | 3utr | S2_FL2,S2_FL3 |
| 3 | 183889010 | *hsa-mir-222* | C | G | *DVL3* | 3utr | S6_FL,S6_tFL |
| 3 | 128199008 | *hsa-mir-586* | T | A | *GATA2* | 3utr | S6_FL,S6_tFL |
| 3 | 49763336 | *hsa-mir-3675* | C | G | *IP6K1* | 3utr | S3_tFL |
| 3 | 128689434 | *hsa-mir-1277* | C | A | *KIAA1257* | 3utr | S2_tFL |
| 3 | 122145587 | *hsa-mir-1290* | G | T | *KPNA1* | 3utr | S5_tFL |
| 3 | 8609402 | *hsa-mir-1229* | G | A | *LMCD1* | 3utr | S5_FL |
| 3 | 197612864 | *hsa-mir-2276* | G | T | *LRCH3* | 3utr | S1_FL,S1_tFL |
| 3 | 120049715 | *hsa-mir-136* | C | G | *LRRC58* | 3utr | S3_tFL |
| 3 | 127409018 | *hsa-mir-1285* | T | G | *MGLL* | 3utr | S3_FL |
| 3 | 122356648 | *hsa-mir-374a* | A | C | *PARP15* | 3utr | S4_tFL |
| 3 | 121150668 | *hsa-mir-138* | C | G | *POLQ* | 3utr | S6_FL,S6_tFL |
| 3 | 87308576 | *hsa-mir-1306* | A | C | *POU1F1* | 3utr | S3_FL,S3_tFL |
| 3 | 133544679 | *hsa-mir-3126* | A | G | *RAB6B* | 3utr | S2_FL2,S2_FL3,S2_tFL |
| 3 | 149679188 | *hsa-mir-200b* | C | T | *RNF13* | 3utr | S2_FL2 |
| 3 | 127800007 | *hsa-mir-1238* | C | A | *RUVBL1* | 3utr | S1_FL,S1_tFL |
| 3 | 170184758 | *hsa-mir-1287* | A | G | *SLC7A14* | 3utr | S2_FL2,S2_FL3,S2_tFL |
| 3 | 156258306 | *hsa-mir-1827* | A | G | *SSR3* | 3utr | S5_FL |
| 3 | 184770109 | *hsa-mir-224* | C | T | *VPS8* | 3utr | S4_FL,S4_tFL |
| 3 | 11667883 | *hsa-mir-1297* | T | G | *VGLL4* | 5utr | S3_FL,S3_tFL |
| 3 | 98250881 | *hsa-mir-1295b* | G | A | *GPR15* | coding | S2_FL2,S2_tFL |
| 3 | 121575963 | *hsa-mir-1270* | T | C | *EAF2* | coding | S2_tFL |
| 3 | 69075204 | *hsa-mir-1255b* | G | A | *TMF1* | coding | S1_tFL |
| 4 | 46923421 | *hsa-mir-5681a* | A | C | *GABRA4* | 3utr | S2_FL2,S2_FL3 |
| 4 | 46927520 | *hsa-mir-3157* | G | A | *GABRA4* | 3utr | S1_tFL |
| 4 | 30732884 | *hsa-mir-495* | A | G | *PCDH7* | 3utr | S1_FL,S1_tFL |
| 4 | 30732983 | *hsa-mir-329* | GTA | G | *PCDH7* | 3utr | S3_tFL |
| 4 | 57801657 | *hsa-mir-3129* | A | T | *REST* | 3utr | S5_FL |
| 4 | 57801952 | *hsa-mir-1279* | A | G | *REST* | 3utr | S2_FL2 |
| 4 | 80823431 | *hsa-mir-208a* | A | C | *ANTXR2* | 3utr | S1_FL,S1_tFL |
| 4 | 75670630 | *hsa-mir-125b* | A | C | *BTC* | 3utr | S2_tFL |
| 4 | 128959426 | *hsa-mir-411* | A | G | *C4orf29* | 3utr | S4_tFL |
| 4 | 103810130 | *hsa-mir-2682* | GCAGA | G | *CISD2* | 3utr | S4_tFL |
| 4 | 73923659 | *hsa-mir-106a* | G | A | *COX18* | 3utr | S1_FL,S1_tFL |
| 4 | 48500769 | *hsa-mir-4423* | C | G | *FRYL* | 3utr | S6_FL |
| 4 | 175560784 | *hsa-mir-7* | G | C | *GLRA3* | 3utr | S3_tFL |
| 4 | 44709057 | *hsa-mir-2054* | G | T | *GNPDA2* | 3utr | S1_FL,S1_tFL |
| 4 | 7061934 | *hsa-mir-4517* | A | G | *GRPEL1* | 3utr | S5_tFL |
| 4 | 156651457 | *hsa-mir-106a* | A | C | *GUCY1A3* | 3utr | S3_tFL |
| 4 | 2231239 | *hsa-mir-1200* | G | T | *HAUS3* | 3utr | S4_FL,S4_tFL |
| 4 | 11398139 | *hsa-mir-6515* | C | T | *HS3ST1* | 3utr | S4_tFL |
| 4 | 55604964 | *hsa-mir-2115* | C | A | ***KIT*** | 3utr | S1_FL,S1_tFL |
| 4 | 88083167 | *hsa-mir-3119* | T | A | *KLHL8* | 3utr | S4_tFL |
| 4 | 54326315 | *hsa-mir-146a* | C | A | *FIP1L1* | 3utr | S3_FL,S3_tFL |
| 4 | 164245890 | *hsa-mir-18a* | GT | G | *NPY1R* | 3utr | S6_FL |
| 4 | 167655217 | *hsa-mir-192* | A | T | *SPOCK3* | 3utr | S2_FL1,S2_tFL |
| 4 | 83825929 | *hsa-mir-615* | G | A | *THAP9* | 3utr | S3_FL,S3_tFL |
| 4 | 164441117 | *hsa-mir-3607* | A | G | *C4orf43* | 3utr | S6_FL,S6_tFL |
| 5 | 132215395 | *hsa-mir-6505* | T | C | *AFF4* | 3utr | S1_FL,S1_tFL |
| 5 | 142604597 | *hsa-mir-4287* | G | A | *ARHGAP26* | 3utr | S6_FL |
| 5 | 151125317 | *hsa-mir-223* | G | C | *ATOX1* | 3utr | S2_FL3 |
| 5 | 148875666 | *hsa-mir-4729* | T | C | *CSNK1A1* | 3utr | S6_FL |
| 5 | 106716920 | *hsa-mir-3667* | G | T | *EFNA5* | 3utr | S2_tFL |
| 5 | 137843353 | *hsa-mir-106a* | T | C | *ETF1* | 3utr | S1_FL,S1_tFL |
| 5 | 108532414 | *hsa-mir-1272* | TTCA | T | *FER* | 3utr | S5_tFL |
| 5 | 160716287 | *hsa-mir-1179* | T | C | *GABRB2* | 3utr | S1_tFL |
| 5 | 37813104 | *hsa-mir-1234* | T | TG | *GDNF* | 3utr | S1_FL,S1_tFL |
| 5 | 179727693 | *hsa-mir-3613* | A | C | *GFPT2* | 3utr | S3_tFL |
| 5 | 132441053 | *hsa-mir-141* | G | T | *HSPA4* | 3utr | S3_tFL |
| 5 | 131818706 | *hsa-mir-192* | T | C | *IRF1* | 3utr | S5_tFL |
| 5 | 50690217 | *hsa-mir-1275* | C | A | *ISL1* | 3utr | S5_FL |
| 5 | 146970782 | *hsa-mir-4286* | G | GT | *JAKMIP2* | 3utr | S1_FL,S1_tFL |
| 5 | 154197002 | *hsa-mir-300* | G | A | *LARP1* | 3utr | S3_tFL |
| 5 | 141533407 | *hsa-let-7a* | G | A | *NDFIP1* | 3utr | S2_tFL |
| 5 | 141323278 | *hsa-mir-1273e* | G | C | *PCDH12* | 3utr | S5_tFL |
| 5 | 140232167 | *hsa-mir-3613* | A | T | *PCDHA9* | 3utr | S6_FL |
| 5 | 149226197 | *hsa-mir-3124* | A | G | *PPARGC1B* | 3utr | S6_FL |
| 5 | 96496744 | *hsa-mir-3653* | A | T | *RIOK2* | 3utr | S3_tFL |
| 5 | 175386586 | *hsa-mir-371a* | A | G | *THOC3* | 3utr | S1_tFL |
| 5 | 87497359 | *hsa-mir-4774* | A | G | *TMEM161B* | 3utr | S1_tFL |
| 5 | 178510529 | *hsa-mir-34b* | G | C | *ZNF354C* | 3utr | S6_FL |
| 5 | 149421364 | *hsa-mir-1184* | G | A | *HMGXB3* | coding | S1_FL,S1_tFL |
| 6 | 35056661 | *hsa-mir-3163* | A | G | *ANKS1A* | 3utr | S2_FL3 |
| 6 | 136582252 | *hsa-mir-126* | TCCTAA | T | ***BCLAF1*** | 3utr | S4_FL |
| 6 | 170103788 | *hsa-mir-1180* | G | A | *C6orf120* | 3utr | S1_FL,S1_tFL |
| 6 | 7586855 | *hsa-mir-3146* | A | G | *DSP* | 3utr | S2_tFL |
| 6 | 132215786 | *hsa-mir-1202* | C | A | *ENPP1* | 3utr | S1_FL,S1_tFL |
| 6 | 24806326 | *hsa-mir-552* | T | C | *FAM65B* | 3utr | S1_FL,S1_tFL |
| 6 | 121769806 | *hsa-mir-3145* | ATATCAT | A | *GJA1* | 3utr | S1_tFL |
| 6 | 26157317 | *hsa-mir-1276* | C | G | *HIST1H1E* | 3utr | S1_FL,S1_tFL |
| 6 | 26123680 | *hsa-mir-3176* | C | G | *HIST1H2BC* | 3utr | S5_tFL |
| 6 | 138663138 | *hsa-mir-1226* | A | G | *KIAA1244* | 3utr | S5_tFL |
| 6 | 39302545 | *hsa-mir-3127* | AGT | A | *KIF6* | 3utr | S3_FL |
| 6 | 74216870 | *hsa-mir-103a* | G | C | *MTO1* | 3utr | S1_FL |
| 6 | 41318457 | *hsa-mir-1225* | T | G | *NCR2* | 3utr | S6_FL |
| 6 | 24146956 | *hsa-mir-182* | T | A | *NRSN1* | 3utr | S6_FL,S6_tFL |
| 6 | 118031596 | *hsa-mir-129* | A | G | *NUS1* | 3utr | S2_tFL |
| 6 | 128290472 | *hsa-mir-1284* | A | T | *PTPRK* | 3utr | S3_tFL |
| 6 | 163989284 | *hsa-mir-1275* | T | C | *QKI* | 3utr | S1_FL,S1_tFL |
| 6 | 153332308 | *hsa-mir-1271* | G | C | *RGS17* | 3utr | S2_FL2,S2_FL3 |
| 6 | 166825162 | *hsa-mir-106a* | C | T | *RPS6KA2* | 3utr | S6_FL |
| 6 | 43639843 | *hsa-mir-3190* | C | T | *RSPH9* | 3utr | S1_FL,S1_tFL |
| 6 | 147889639 | *hsa-mir-106a* | G | T | *SAMD5* | 3utr | S3_FL,S3_tFL |
| 6 | 25783234 | *hsa-mir-197* | A | C | *SLC17A1* | 3utr | S6_FL,S6_tFL |
| 6 | 21598724 | *hsa-mir-361* | GA | G | ***SOX4*** | 3utr | S3_FL,S3_tFL |
| 6 | 86267774 | *hsa-mir-369* | T | C | *SNX14* | 3utr | S6_FL |
| 6 | 158519568 | *hsa-mir-2355* | T | G | *SYNJ2* | 3utr | S3_FL |
| 6 | 35442031 | *hsa-mir-22* | A | G | *TEAD3* | 3utr | S1_FL,S1_tFL |
| 6 | 50811115 | *hsa-mir-1237* | A | T | *TFAP2B* | 3utr | S3_tFL |
| 6 | 30153021 | *hsa-mir-1224* | T | C | *TRIM26* | 3utr | S1_FL,S1_tFL |
| 6 | 25983973 | *hsa-mir-18a* | T | G | *TRIM38* | 3utr | S2_FL3 |
| 6 | 167771419 | *hsa-mir-4496* | T | G | *TCP10* | 3utr | S6_FL |
| 6 | 30692754 | *hsa-mir-1302* | C | CTT | *TUBB* | 3utr | S3_FL |
| 6 | 110421523 | *hsa-mir-30a* | A | G | *WASF1* | 3utr | S4_FL,S4_tFL |
| 6 | 28402556 | *hsa-mir-1245b* | A | C | *ZSCAN23* | 3utr | S3_tFL |
| 7 | 106299866 | *hsa-mir-3685* | A | G | *RP5-892G19.1* | 3utr | S5_tFL |
| 7 | 106299908 | *hsa-mir-28* | T | A | *RP5-892G19.1* | 3utr | S5_tFL |
| 7 | 19735117 | *hsa-mir-3140* | T | C | ***TWISTNB*** | 3utr | S2_FL1,S2_FL2,S2_FL3,S2_tFL |
| 7 | 19735141 | *hsa-mir-1267* | A | G | ***TWISTNB*** | 3utr | S2_FL1,S2_FL2,S2_FL3,S2_tFL |
| 7 | 150558458 | *hsa-mir-654* | G | A | *ABP1* | 3utr | S4_FL,S4_tFL |
| 7 | 102739179 | *hsa-mir-222* | A | G | *ARMC10* | 3utr | S5_FL |
| 7 | 55278583 | *hsa-mir-1295b* | C | T | *EGFR* | 3utr | S5_tFL |
| 7 | 22982689 | *hsa-mir-593* | C | G | *FAM126A* | 3utr | S2_FL1,S2_FL2,S2_FL3,S2_tFL |
| 7 | 102449275 | *hsa-mir-1293* | A | C | *FAM185A* | 3utr | S4_tFL |
| 7 | 76824492 | *hsa-mir-3162* | A | C | *FGL2* | 3utr | S6_FL |
| 7 | 92086889 | *hsa-mir-3191* | G | C | *GATAD1* | 3utr | S1_tFL |
| 7 | 935183 | *hsa-mir-3195* | G | A | *GET4* | 3utr | S5_FL |
| 7 | 93539407 | *hsa-mir-135a* | G | C | *GNGT1* | 3utr | S6_FL |
| 7 | 50659032 | *hsa-mir-127* | CTAGGCTA.. | C | *GRB10* | 3utr | S1_FL |
| 7 | 27202235 | *hsa-mir-376a* | T | A | ***HOXA9*** | 3utr | S2_tFL |
| 7 | 1510196 | *hsa-mir-4443* | G | A | *INTS1* | 3utr | S4_FL |
| 7 | 66106891 | *hsa-mir-3614* | G | C | *KCTD7* | 3utr | S1_tFL |
| 7 | 127897316 | *hsa-mir-520d* | T | C | *LEP* | 3utr | S6_FL |
| 7 | 156473795 | *hsa-mir-548ae* | T | A | *LMBR1* | 3utr | S6_FL |
| 7 | 1580362 | *hsa-mir-1227* | G | T | ***MAFK*** | 3utr | S3_FL |
| 7 | 45002610 | *hsa-mir-1226* | G | A | *MYO1G* | 3utr | S5_tFL |
| 7 | 44425397 | *hsa-mir-4691* | C | T | *NUDCD3* | 3utr | S1_tFL |
| 7 | 94296494 | *hsa-mir-561* | C | T | *PEG10* | 3utr | S5_FL |
| 7 | 131812650 | *hsa-mir-802* | C | T | *PLXNA4* | 3utr | S3_tFL |
| 7 | 72419066 | *hsa-mir-4311* | C | CTG | *POM121* | 3utr | S3_FL,S4_tFL |
| 7 | 141537926 | *hsa-mir-1299* | G | A | *PRSS37* | 3utr | S1_tFL |
| 7 | 44920885 | *hsa-mir-323b* | T | C | *PURB* | 3utr | S4_tFL |
| 7 | 80373350 | *hsa-mir-200b* | T | A | *SEMA3C* | 3utr | S6_tFL |
| 7 | 84625565 | *hsa-mir-5701* | T | G | *SEMA3D* | 3utr | S5_FL |
| 7 | 17922385 | *hsa-mir-200a* | G | A | *SNX13* | 3utr | S3_tFL |
| 7 | 16501292 | *hsa-mir-330* | AT | A | *SOSTDC1* | 3utr | S1_FL,S1_tFL |
| 7 | 97845855 | *hsa-mir-199a* | T | C | *TECPR1* | 3utr | S6_FL,S6_tFL |
| 7 | 128595317 | *hsa-mir-1289* | T | C | *TNPO3* | 3utr | S1_FL,S1_tFL |
| 7 | 6200245 | *hsa-mir-4797* | A | T | *USP42* | 3utr | S1_tFL |
| 7 | 120980591 | *hsa-mir-4719* | A | T | *WNT16* | 3utr | S1_FL,S1_tFL |
| 7 | 148508727 | *hsa-mir-144* | T | A | ***EZH2*** | coding | S1_FL,S1_tFL,S2_FL1,S2_FL2,S2_FL3,S2_tFL |
| 7 | 148508764 | *hsa-mir-4793* | T | C | ***EZH2*** | coding | S2_FL1,S2_FL2,S2_FL3,S2_tFL |
| 7 | 13935600 | *hsa-mir-3657* | A | T | ***ETV1*** | coding | S1_FL,S1_tFL |
| 7 | 151927358 | *hsa-mir-153* | T | TA | ***MLL3*** | coding | S3_tFL |
| 8 | 87480573 | *hsa-mir-376a* | G | A | *FAM82B* | 3utr | S5_tFL |
| 8 | 87486431 | *hsa-mir-224* | C | T | *FAM82B* | 3utr | S2_FL2,S2_FL3 |
| 8 | 121244027 | *hsa-mir-1290* | C | A | *COL14A1* | 3utr | S3_tFL |
| 8 | 117657097 | *hsa-mir-135a* | G | A | *EIF3H* | 3utr | S1_FL,S1_tFL |
| 8 | 139142643 | *hsa-mir-3163* | T | TA | *FAM135B* | 3utr | S2_FL2,S2_FL3,S2_tFL |
| 8 | 11280997 | *hsa-mir-4436b* | C | G | *FAM167A* | 3utr | S1_tFL |
| 8 | 80676728 | *hsa-mir-3129* | T | A | *HEY1* | 3utr | S3_tFL |
| 8 | 75147151 | *hsa-mir-101* | A | G | *JPH1* | 3utr | S6_FL,S6_tFL |
| 8 | 99443066 | *hsa-mir-4635* | C | G | *KCNS2* | 3utr | S3_tFL |
| 8 | 125580414 | *hsa-mir-1255b* | C | T | *NDUFB9* | 3utr | S4_FL,S4_tFL |
| 8 | 90938856 | *hsa-mir-410* | T | G | *OSGIN2* | 3utr | S5_tFL |
| 8 | 52731707 | *hsa-mir-3689a* | A | G | *PCMTD1* | 3utr | S3_FL |
| 8 | 66634574 | *hsa-mir-4762* | C | T | *PDE7A* | 3utr | S1_FL |
| 8 | 86573416 | *hsa-mir-105* | A | G | *REXO1L1* | 3utr | S4_tFL |
| 8 | 105907686 | *hsa-mir-3692* | G | T | *RP11-127H5.1* | 3utr | S6_FL,S6_tFL |
| 8 | 42692834 | *hsa-mir-548as* | C | A | *THAP1* | 3utr | S5_tFL |
| 8 | 59361626 | *hsa-mir-1178* | A | G | *UBXN2B* | 3utr | S5_tFL |
| 8 | 144357600 | *hsa-mir-4632* | C | G | *GLI4* | 3utr | S2_FL1,S2_FL2,S2_FL3,S2_tFL |
| 8 | 18430069 | *hsa-mir-1207* | A | T | *PSD3* | coding | S2_tFL |
| 9 | 78808412 | *hsa-mir-4287* | A | C | *PCSK5* | 3utr | S2_tFL |
| 9 | 78975278 | *hsa-mir-5009* | A | T | *PCSK5* | 3utr | S4_tFL |
| 9 | 33441702 | *hsa-mir-146b* | A | AC | *AQP3* | 3utr | S6_FL |
| 9 | 15972581 | *hsa-mir-1292* | C | A | *C9orf93* | 3utr | S1_FL,S1_tFL |
| 9 | 80887336 | *hsa-mir-4299* | A | G | *CEP78* | 3utr | S2_tFL |
| 9 | 1056564 | *hsa-mir-382* | T | G | *DMRT2* | 3utr | S2_FL1,S2_FL2,S2_FL3,S2_tFL |
| 9 | 111936168 | *hsa-mir-1290* | A | G | *EPB41L4B* | 3utr | S6_FL |
| 9 | 98730787 | *hsa-mir-185* | A | C | *C9orf102* | 3utr | S1_FL,S1_tFL |
| 9 | 3827358 | *hsa-mir-3612* | A | T | *GLIS3* | 3utr | S4_FL,S4_tFL |
| 9 | 104331953 | *hsa-mir-186* | A | G | *GRIN3A* | 3utr | S2_FL3 |
| 9 | 115232919 | *hsa-mir-1277* | A | G | *HSDL2* | 3utr | S5_FL |
| 9 | 132996079 | *hsa-mir-3200* | T | G | *NCS1* | 3utr | S1_tFL |
| 9 | 36840138 | *hsa-mir-4264* | C | G | ***PAX5*** | 3utr | S3_FL,S3_tFL |
| 9 | 112139908 | *hsa-mir-199a* | G | C | *PTPN3* | 3utr | S3_tFL |
| 9 | 135138179 | *hsa-mir-1245b* | A | C | *SETX* | 3utr | S2_FL1,S2_FL2,S2_FL3,S2_tFL |
| 9 | 33043722 | *hsa-mir-4316* | G | GA | *SMU1* | 3utr | S1_FL,S1_tFL |
| 9 | 93659684 | *hsa-mir-3064* | A | G | *SYK* | 3utr | S2_FL2,S2_FL3 |
| 9 | 104237051 | *hsa-mir-185* | C | G | *C9orf125* | 3utr | S1_tFL |
| 9 | 135767598 | *hsa-mir-1178* | C | A | *TSC1* | 3utr | S3_tFL |
| 9 | 139978744 | *hsa-mir-1321* | A | G | *UAP1L1* | 3utr | S6_tFL |
| 9 | 34658640 | *hsa-mir-217* | G | A | *IL11RA* | coding | S2_FL1,S2_FL2,S2_FL3,S2_tFL |
| 9 | 100410462 | *hsa-mir-1256* | C | G | *NCBP1* | coding | S1_tFL |
| 9 | 102722410 | *hsa-mir-185* | G | T | *STX17* | coding | S1_FL,S1_tFL |
| 10 | 5807657 | *hsa-mir-15a* | A | C | *GDI2* | 3utr | S5_tFL |
| 10 | 5807705 | *hsa-mir-1231* | A | G | *GDI2* | 3utr | S5_tFL |
| 10 | 5807722 | *hsa-mir-33a* | A | C | *GDI2* | 3utr | S5_tFL |
| 10 | 101611732 | *hsa-mir-1910* | A | G | *ABCC2* | 3utr | S6_FL |
| 10 | 75881949 | *hsa-mir-424* | T | C | *AP3M1* | 3utr | S5_FL,S5_tFL |
| 10 | 104623993 | *hsa-mir-3682* | C | T | *C10orf32* | 3utr | S2_FL2 |
| 10 | 11376834 | *hsa-mir-4254* | G | A | *CELF2* | 3utr | S1_FL,S1_tFL |
| 10 | 73768236 | *hsa-mir-3184* | C | T | *CHST3* | 3utr | S4_FL,S4_tFL |
| 10 | 70744144 | *hsa-mir-4670* | C | T | *DDX21* | 3utr | S3_tFL |
| 10 | 25273615 | *hsa-mir-4307* | GT | G | *ENKUR* | 3utr | S1_tFL |
| 10 | 90773889 | *hsa-mir-140* | T | TA | ***FAS*** | 3utr | S2_FL3 |
| 10 | 113916242 | *hsa-mir-103a* | T | A | *GPAM* | 3utr | S4_FL,S4_tFL |
| 10 | 1063493 | *hsa-mir-3676* | G | C | *GTPBP4* | 3utr | S5_FL,S5_tFL |
| 10 | 69682490 | *hsa-mir-218* | C | A | *HERC4* | 3utr | S6_FL,S6_tFL |
| 10 | 27963059 | *hsa-mir-5700* | C | T | *MKX* | 3utr | S1_tFL |
| 10 | 88696329 | *hsa-mir-1248* | G | C | *MMRN2* | 3utr | S1_tFL |
| 10 | 115541346 | *hsa-mir-3122* | A | C | *C10orf81* | 3utr | S6_FL |
| 10 | 118236708 | *hsa-mir-3190* | A | G | *PNLIPRP3* | 3utr | S6_FL |
| 10 | 133772780 | *hsa-mir-3191* | G | C | *PPP2R2D* | 3utr | S2_tFL |
| 10 | 54057140 | *hsa-mir-633* | T | TA | *PRKG1* | 3utr | S2_tFL |
| 10 | 98760081 | *hsa-mir-1245b* | T | C | *ARHGAP19* | 3utr | S2_FL1,S2_FL2,S2_FL3,S2_tFL |
| 10 | 107023790 | *hsa-mir-33a* | C | T | *SORCS3* | 3utr | S4_FL,S4_tFL |
| 10 | 25314525 | *hsa-mir-4653* | G | T | *THNSL1* | 3utr | S1_FL,S1_tFL |
| 11 | 2317556 | *hsa-mir-3064* | A | T | *C11orf21* | 3utr | S3_FL |
| 11 | 131529982 | *hsa-mir-4457* | C | T | *C11orf39* | 3utr | S1_FL,S1_tFL |
| 11 | 109296866 | *hsa-mir-217* | T | C | *C11orf87* | 3utr | S1_tFL |
| 11 | 111405233 | *hsa-mir-2115* | A | G | *C11orf88* | 3utr | S1_tFL |
| 11 | 34122120 | *hsa-mir-758* | T | G | *CAPRIN1* | 3utr | S6_FL,S6_tFL |
| 11 | 60894894 | *hsa-mir-1914* | C | A | ***CD5*** | 3utr | S6_FL |
| 11 | 94863498 | *hsa-mir-3136* | TG | T | *ENDOD1* | 3utr | S2_tFL |
| 11 | 33762796 | *hsa-mir-4255* | T | C | *FBXO3* | 3utr | S3_tFL |
| 11 | 72548986 | *hsa-mir-4263* | A | C | *FCHSD2* | 3utr | S3_tFL |
| 11 | 27018026 | *hsa-mir-3661* | T | A | *FIBIN* | 3utr | S3_FL |
| 11 | 1756496 | *hsa-mir-1271* | G | A | *IFITM10* | 3utr | S2_FL1,S2_FL2,S2_FL3,S2_tFL |
| 11 | 77635688 | *hsa-mir-4484* | A | G | *INTS4* | 3utr | S1_tFL |
| 11 | 28353434 | *hsa-mir-4313* | G | A | *METTL15* | 3utr | S4_tFL |
| 11 | 2966359 | *hsa-mir-1273f* | A | T | *NAP1L4* | 3utr | S5_tFL |
| 11 | 132288788 | *hsa-mir-128* | C | G | *OPCML* | 3utr | S5_tFL |
| 11 | 4702538 | *hsa-mir-1292* | C | T | *OR51E2* | 3utr | S5_FL,S5_tFL |
| 11 | 12550853 | *hsa-mir-4700* | C | T | *PARVA* | 3utr | S2_tFL |
| 11 | 18749965 | *hsa-mir-1237* | A | G | *PTPN5* | 3utr | S4_FL,S4_tFL |
| 11 | 65485939 | *hsa-mir-3616* | A | C | *RNASEH2C* | 3utr | S1_FL,S1_tFL |
| 11 | 15993470 | *hsa-mir-4484* | T | C | *SOX6* | 3utr | S5_FL |
| 11 | 124567404 | *hsa-mir-203a* | A | C | *SPA17* | 3utr | S6_FL,S6_tFL |
| 11 | 57505281 | *hsa-mir-1321* | A | C | *TMX2* | 3utr | S6_tFL |
| 11 | 4620424 | *hsa-mir-214* | T | A | *TRIM68* | 3utr | S2_FL2,S2_FL3 |
| 11 | 3380000 | *hsa-mir-1915* | T | TCTATTCCTT… | *ZNF195* | 3utr | S2_tFL |
| 11 | 1912020 | *hsa-mir-4704* | C | G | *C11orf89* | 5utr | S4_tFL |
| 11 | 66130796 | *hsa-mir-1915* | G | C | *RP11-867G23.8* | coding | S6_FL |
| 11 | 66130901 | *hsa-mir-1254* | G | A | *RP11-867G23.8* | coding | S2_FL1,S2_FL2,S2_tFL |
| 12 | 42480760 | *hsa-mir-1229* | A | C | *GXYLT1* | 3utr | S2_tFL |
| 12 | 42480767 | *hsa-mir-181c* | A | G | *GXYLT1* | 3utr | S2_tFL |
| 12 | 42480798 | *hsa-mir-320a* | C | A | *GXYLT1* | 3utr | S2_tFL |
| 12 | 42480799 | *hsa-mir-320a* | G | A | *GXYLT1* | 3utr | S2_tFL |
| 12 | 110606884 | *hsa-mir-1252* | T | C | *IFT81* | 3utr | S6_FL,S6_tFL |
| 12 | 110606885 | *hsa-mir-1252* | C | T | *IFT81* | 3utr | S6_FL,S6_tFL |
| 12 | 110606888 | *hsa-mir-3125* | C | T | *IFT81* | 3utr | S2_FL3 |
| 12 | 110606889 | *hsa-mir-3125* | T | C | *IFT81* | 3utr | S2_FL3 |
| 12 | 27575606 | *hsa-mir-141* | GT | G | *ARNTL2* | 3utr | S3_FL,S3_tFL |
| 12 | 70048260 | *hsa-mir-135a* | A | G | *BEST3* | 3utr | S1_FL,S1_tFL |
| 12 | 25146497 | *hsa-mir-1261* | A | G | *C12orf77* | 3utr | S1_FL,S1_tFL |
| 12 | 54626806 | *hsa-mir-29a* | T | A | *CBX5* | 3utr | S3_tFL |
| 12 | 63953768 | *hsa-mir-1303* | T | C | *DPY19L2* | 3utr | S3_tFL |
| 12 | 50261026 | *hsa-mir-296* | G | A | *FAIM2* | 3utr | S3_FL,S3_tFL |
| 12 | 51747877 | *hsa-mir-146b* | G | T | *GALNT6* | 3utr | S1_FL,S1_tFL |
| 12 | 13068992 | *hsa-mir-1263* | C | T | *GPRC5A* | 3utr | S6_FL |
| 12 | 14930580 | *hsa-mir-3129* | T | C | *H2AFJ* | 3utr | S2_tFL |
| 12 | 14923694 | *hsa-mir-1289* | C | G | *HIST4H4* | 3utr | S5_tFL |
| 12 | 54405190 | *hsa-mir-1234* | C | T | *HOXC8* | 3utr | S3_tFL |
| 12 | 393639 | *hsa-mir-3166* | T | C | *KDM5A* | 3utr | S3_FL,S3_tFL |
| 12 | 88889607 | *hsa-mir-3133* | A | G | ***KITLG*** | 3utr | S1_FL,S1_tFL |
| 12 | 52862416 | *hsa-mir-2115* | C | T | *KRT6C* | 3utr | S1_FL,S1_tFL |
| 12 | 31821887 | *hsa-mir-183* | G | A | *METTL20* | 3utr | S1_tFL |
| 12 | 81102767 | *hsa-mir-141* | C | T | *MYF6* | 3utr | S4_FL |
| 12 | 86276428 | *hsa-mir-943* | T | G | *NTS* | 3utr | S6_FL,S6_tFL |
| 12 | 50517503 | *hsa-mir-1273g* | A | T | *AC074032.1* | 3utr | S1_FL |
| 12 | 21622061 | *hsa-mir-103b* | A | G | *PYROXD1* | 3utr | S4_tFL |
| 12 | 65089357 | *hsa-mir-3140* | G | A | *RASSF3* | 3utr | S6_tFL |
| 12 | 48438318 | *hsa-mir-196a* | A | G | *SENP1* | 3utr | S5_tFL |
| 12 | 105198038 | *hsa-mir-3653* | A | G | *SLC41A2* | 3utr | S6_FL |
| 12 | 299853 | *hsa-mir-34a* | A | G | *SLC6A12* | 3utr | S2_FL1,S2_FL2,S2_FL3,S2_tFL |
| 12 | 21421596 | *hsa-mir-3153* | G | A | *SLCO1A2* | 3utr | S1_FL |
| 12 | 23682820 | *hsa-mir-138* | C | T | *SOX5* | 3utr | S1_FL,S1_tFL |
| 12 | 11285839 | *hsa-mir-3130* | A | T | *TAS2R30* | 3utr | S3_tFL |
| 12 | 29659238 | *hsa-mir-129* | CT | C | *TMTC1* | 3utr | S2_tFL |
| 12 | 109972748 | *hsa-mir-512* | G | A | *UBE3B* | 3utr | S1_tFL |
| 12 | 6572216 | *hsa-mir-4290* | C | T | *VAMP1* | 3utr | S5_tFL |
| 12 | 48237047 | *hsa-mir-211* | C | T | *VDR* | 3utr | S3_FL,S3_tFL |
| 12 | 57395205 | *hsa-let-7a* | G | A | *ZBTB39* | 3utr | S2_FL2,S2_FL3 |
| 12 | 18644413 | *hsa-mir-346* | T | A | *PIK3C* | coding | S2_FL2,S2_FL3 |
| 12 | 85255524 | *hsa-mir-3664* | T | G | *SLC6A15* | coding | S1_FL,S1_tFL |
| 13 | 50205385 | *hsa-mir-126* | C | A | *ARL11* | 3utr | S5_FL |
| 13 | 41132162 | *hsa-mir-2861* | C | T | *FOXO1* | 3utr | S1_tFL |
| 13 | 30777622 | *hsa-mir-106a* | A | G | *KATNAL1* | 3utr | S4_tFL |
| 13 | 113751454 | *hsa-mir-1827* | G | A | *MCF2L* | 3utr | S5_tFL |
| 13 | 97637989 | *hsa-mir-340* | A | G | *OXGR1* | 3utr | S6_FL |
| 13 | 58302443 | *hsa-mir-130a* | T | C | *PCDH17* | 3utr | S1_FL,S1_tFL |
| 13 | 51530854 | *hsa-mir-2053* | A | T | *RNASEH2B* | 3utr | S6_FL |
| 13 | 79189635 | *hsa-mir-3149* | T | G | *RNF219* | 3utr | S3_FL,S3_tFL |
| 13 | 27641060 | *hsa-mir-4762* | T | C | *USP12* | 3utr | S5_tFL |
| 14 | 105213399 | *hsa-mir-134* | G | C | *ADSSL1* | 3utr | S1_tFL |
| 14 | 99639429 | *hsa-mir-299* | A | T | ***BCL11B*** | 3utr | S2_FL2,S2_FL3 |
| 14 | 54416456 | *hsa-mir-4724* | T | G | *BMP4* | 3utr | S1_FL,S1_tFL |
| 14 | 80665370 | *hsa-mir-5196* | A | C | *DIO2* | 3utr | S1_tFL |
| 14 | 100604645 | *hsa-mir-2467* | G | A | ***EVL*** | 3utr | S5_FL |
| 14 | 45543318 | *hsa-mir-3668* | A | G | *FAM179B* | 3utr | S5_FL |
| 14 | 86094878 | *hsa-mir-1238* | G | A | *FLRT2* | 3utr | S3_FL,S3_tFL |
| 14 | 93404640 | *hsa-mir-1915* | C | A | *ITPK1* | 3utr | S3_tFL |
| 14 | 57877459 | *hsa-mir-3658* | A | T | *NAA30* | 3utr | S5_FL |
| 14 | 77732256 | *hsa-mir-4632* | C | T | *NGB* | 3utr | S2_tFL |
| 14 | 37149553 | *hsa-mir-4709* | A | G | *SLC25A21* | 3utr | S2_FL2,S2_FL3,S2_tFL |
| 14 | 70839142 | *hsa-mir-1229* | C | T | *SYNJ2BP* | 3utr | S5_tFL |
| 14 | 92246290 | *hsa-mir-1277* | T | A | *TC2N* | 3utr | S4_tFL |
| 14 | 67938137 | *hsa-mir-4261* | A | C | *TMEM229B* | 3utr | S1_FL,S1_tFL |
| 14 | 89343803 | *hsa-mir-512* | GA | G | *TTC8* | 3utr | S2_tFL |
| 15 | 100255955 | *hsa-mir-3613* | A | C | *AC022692.1* | 3utr | S2_FL2,S2_FL3 |
| 15 | 78463428 | *hsa-mir-155* | G | A | *ACSBG1* | 3utr | S1_FL,S1_tFL |
| 15 | 80253404 | *hsa-mir-122* | T | C | ***BCL2A1*** | 3utr | S2_tFL |
| 15 | 78916746 | *hsa-mir-146b* | G | A | *CHRNB4* | 3utr | S2_FL1,S2_FL2,S2_FL3,S2_tFL |
| 15 | 35044128 | *hsa-mir-518a* | A | T | *GJD2* | 3utr | S1_tFL |
| 15 | 59500519 | *hsa-mir-410* | TTA | T | *LDHAL6B* | 3utr | S4_tFL |
| 15 | 88403334 | *hsa-mir-380* | G | T | *NTRK3* | 3utr | S3_FL,S3_tFL |
| 15 | 77401915 | *hsa-mir-133a* | G | C | *AC087465.1* | 3utr | S3_tFL |
| 15 | 63555876 | *hsa-mir-1262* | A | C | *RAB8B* | 3utr | S6_FL,S6_tFL |
| 15 | 60787016 | *hsa-mir-4318* | C | T | *R****ORA*** | 3utr | S1_FL,S1_tFL |
| 15 | 72433236 | *hsa-mir-1179* | T | C | *SENP8* | 3utr | S2_FL1,S2_FL2,S2_FL3,S2_tFL |
| 15 | 75949584 | *hsa-mir-17* | C | T | *SNX33* | 3utr | S1_tFL |
| 15 | 70340941 | *hsa-mir-4715* | C | T | *TLE3* | 3utr | S5_tFL |
| 15 | 91541842 | *hsa-mir-3137* | G | A | *VPS33B* | 3utr | S4_FL,S4_tFL |
| 15 | 42708764 | *hsa-mir-3529* | G | A | *ZFP106* | 3utr | S5_tFL |
| 15 | 25940037 | *hsa-mir-1307* | G | A | *ATP10A* | coding | S5_tFL |
| 15 | 25958929 | *hsa-mir-184* | G | A | *ATP10A* | coding | S3_FL,S3_tFL |
| 15 | 25958933 | *hsa-mir-184* | G | A | *ATP10A* | coding | S1_FL,S1_tFL |
| 15 | 45249109 | *hsa-mir-181b* | C | T | *C15orf43* | coding | S1_FL,S1_tFL |
| 15 | 23686239 | *hsa-mir-1236* | T | C | *GOLGA6L2* | coding | S4_FL |
| 16 | 29870063 | *hsa-mir-1286* | C | T | *CDIPT* | 3utr | S6_tFL |
| 16 | 10623868 | *hsa-mir-4471* | G | T | *EMP2* | 3utr | S3_FL,S3_tFL |
| 16 | 23476271 | *hsa-mir-1269a* | CACACTTA… | C | *GGA2* | 3utr | S5_tFL |
| 16 | 29917422 | *hsa-mir-1207* | A | C | *ASPHD1* | 3utr | S2_FL2 |
| 16 | 55617432 | *hsa-mir-10b* | T | C | *LPCAT2* | 3utr | S1_FL,S1_tFL |
| 16 | 69736839 | *hsa-mir-30a* | C | G | *NFAT5* | 3utr | S3_tFL |
| 16 | 50263455 | *hsa-mir-4446* | A | G | *PAPD5* | 3utr | S6_tFL |
| 16 | 3022129 | *hsa-mir-1207* | C | T | *PAQR4* | 3utr | S2_FL1,S2_FL2,S2_FL3,S2_tFL |
| 16 | 21995228 | *hsa-mir-1278* | C | CA | *PDZD9* | 3utr | S5_tFL |
| 16 | 31142966 | *hsa-mir-4436b* | T | C | *PRSS8* | 3utr | S5_FL,S5_tFL |
| 16 | 18819330 | *hsa-mir-3150a* | GC | G | *SMG1* | 3utr | S2_FL1,S2_FL2,S2_tFL |
| 16 | 2569676 | *hsa-mir-1307* | T | C | *ATP6V0C* | coding | S4_FL,S4_tFL |
| 16 | 2569708 | *hsa-mir-1226* | T | C | *ATP6V0C* | coding | S4_FL,S4_tFL |
| 16 | 2569631 | *hsa-mir-1228* | T | A | *ATP6V0C* | coding | S4_FL,S4_tFL |
| 17 | 9818018 | *hsa-mir-3133* | T | A | *GAS7* | 3utr | S3_tFL |
| 17 | 9820289 | *hsa-mir-3942* | C | A | *GAS7* | 3utr | S3_FL,S3_tFL |
| 17 | 76849749 | *hsa-mir-105* | A | C | *TIMP2* | 3utr | S3_FL |
| 17 | 66864089 | *hsa-mir-1295b* | G | A | *ABCA8* | 3utr | S3_tFL |
| 17 | 34198769 | *hsa-mir-758* | G | A | *CCL5* | 3utr | S1_tFL |
| 17 | 72608386 | *hsa-mir-3120* | G | C | *CD300E* | 3utr | S2_FL1 |
| 17 | 7485372 | *hsa-mir-153* | A | T | *CD68* | 3utr | S6_FL |
| 17 | 77078803 | *hsa-mir-1228* | A | C | *ENGASE* | 3utr | S2_tFL |
| 17 | 80553855 | *hsa-mir-345* | G | A | *FOXK2* | 3utr | S1_FL,S1_tFL |
| 17 | 9793627 | *hsa-mir-2278* | G | A | *GLP2R* | 3utr | S2_FL1,S2_FL2,S2_FL3,S2_tFL |
| 17 | 74710001 | *hsa-mir-1291* | G | A | *JMJD6* | 3utr | S6_tFL |
| 17 | 61771305 | *hsa-mir-1184* | G | T | *MAP3K3* | 3utr | S3_FL,S3_tFL |
| 17 | 16679597 | *hsa-mir-3686* | A | T | *CCDC144A* | 3utr | S5_tFL |
| 17 | 62316 | *hsa-mir-4289* | C | T | *RPH3AL* | 3utr | S6_FL |
| 17 | 27282195 | *hsa-mir-3150b* | A | C | *SEZ6* | 3utr | S2_FL1,S2_FL2,S2_FL3,S2_tFL |
| 17 | 33594728 | *hsa-mir-139* | G | T | *SLFN5* | 3utr | S1_tFL |
| 17 | 38783119 | *hsa-mir-4649* | G | T | *SMARCE1* | 3utr | S5_FL |
| 17 | 18167760 | *hsa-mir-4285* | C | T | *SMCR7* | 3utr | S6_FL,S6_tFL |
| 17 | 36687237 | *hsa-mir-1286* | A | G | *SRCIN1* | 3utr | S1_FL |
| 17 | 7221413 | *hsa-mir-432* | G | A | *GPS2* | 5utr | S3_tFL |
| 17 | 37933932 | *hsa-mir-1256* | TC | T | *IKZF3* | coding | S1_FL |
| 17 | 37933935 | *hsa-mir-1256* | T | G | *IKZF3* | coding | S1_FL |
| 17 | 37933940 | *hsa-mir-19a* | C | G | *IKZF3* | coding | S1_FL |
| 17 | 76851902 | *hsa-mir-520a* | G | A | *TIMP2* | coding | S5_FL,S5_tFL |
| 17 | 40948320 | *hsa-mir-3921* | GCT | G | ***WNK4*** | coding | S4_tFL |
| 17 | 8789893 | *hsa-mir-939* | C | T | *PIK3R5* | coding | S4_FL,S4_tFL |
| 18 | 60793448 | *hsa-mir-5008* | G | AGGAGAG | ***BCL2*** | 3utr | S3_FL |
| 18 | 60793447 | *hsa-mir-5008* | G | A | ***BCL2*** | 3utr | S3_FL |
| 18 | 60793641 | *hsa-mir-6071* | A | C | ***BCL2*** | 3utr | S3_tFL |
| 18 | 60793653 | *hsa-mir-138* | T | C | ***BCL2*** | 3utr | S3_FL,S3_tFL |
| 18 | 60793906 | *hsa-mir-27a* | G | A | ***BCL2*** | 3utr | S3_FL,S3_tFL |
| 18 | 47349754 | *hsa-mir-101* | C | T | *MYO5B* | 3utr | S4_tFL |
| 18 | 47350291 | *hsa-mir-1323* | C | A | *MYO5B* | 3utr | S2_tFL |
| 18 | 47350304 | *hsa-mir-144* | C | T | *MYO5B* | 3utr | S4_tFL |
| 18 | 47350307 | *hsa-mir-144* | G | A | *MYO5B* | 3utr | S4_tFL |
| 18 | 47350444 | *hsa-mir-4311* | A | G | *MYO5B* | 3utr | S4_tFL |
| 18 | 47350494 | *hsa-mir-433* | A | G | *MYO5B* | 3utr | S4_tFL |
| 18 | 47350459 | *hsa-mir-3140* | C | CTTTATGT | *MYO5B* | 3utr | S4_tFL |
| 18 | 47352742 | *hsa-mir-216b* | T | G | *MYO5B* | 3utr | S1_tFL |
| 18 | 47352754 | *hsa-mir-2681* | A | G | *MYO5B* | 3utr | S1_tFL |
| 18 | 40848310 | *hsa-mir-3606* | A | T | *SYT4* | 3utr | S1_FL,S1_tFL |
| 18 | 40848626 | *hsa-mir-1294* | G | T | *SYT4* | 3utr | S1_FL,S1_tFL |
| 18 | 40848861 | *hsa-mir-371a* | A | C | *SYT4* | 3utr | S1_FL,S1_tFL |
| 18 | 63531068 | *hsa-mir-150* | T | A | *CDH7* | 3utr | S3_FL,S3_tFL |
| 18 | 67509881 | *hsa-mir-1272* | A | T | *DOK6* | 3utr | S1_FL |
| 18 | 11881569 | *hsa-mir-3202* | C | G | *GNAL* | 3utr | S1_tFL |
| 18 | 70411236 | *hsa-mir-154* | A | G | *NETO1* | 3utr | S2_FL1,S2_FL3,S2_tFL |
| 18 | 21743050 | *hsa-mir-335* | T | A | *OSBPL1A* | 3utr | S1_FL,S1_tFL |
| 18 | 43422407 | *hsa-mir-342* | T | A | *SIGLEC15* | 3utr | S3_FL |
| 18 | 72907534 | *hsa-mir-1229* | T | C | *ZADH2* | 3utr | S6_FL |
| 18 | 9815126 | *hsa-mir-4659a* | C | T | *RAB31* | coding | S1_tFL |
| 18 | 9887493 | *hsa-mir-2110* | T | C | *TXNDC2* | coding | S6_FL |
| 19 | 55899833 | *hsa-mir-4761* | G | A | *RPL28* | 3utr | S3_tFL |
| 19 | 55903097 | *hsa-mir-4265* | A | G | *RPL28* | 3utr | S2_FL1,S2_FL2,S2_FL3,S2_tFL |
| 19 | 44886986 | *hsa-mir-149* | A | ATCAAAT | *ZNF285* | 3utr | S3_FL |
| 19 | 44890193 | *hsa-mir-1225* | T | C | *ZNF285* | 3utr | S6_FL |
| 19 | 15360063 | *hsa-mir-1299* | C | T | *BRD4* | 3utr | S3_tFL |
| 19 | 541881 | *hsa-mir-132* | G | A | *CDC34* | 3utr | S5_FL |
| 19 | 42311427 | *hsa-mir-4797* | T | C | *CEACAM3* | 3utr | S5_FL |
| 19 | 14845672 | *hsa-mir-4438* | C | T | *EMR2* | 3utr | S1_FL,S1_tFL |
| 19 | 46623584 | *hsa-mir-346* | T | G | *IGFL3* | 3utr | S6_tFL |
| 19 | 54744436 | *hsa-mir-139* | G | A | *LILRA6* | 3utr | S5_FL |
| 19 | 17323594 | *hsa-mir-140* | T | C | *MYO9B* | 3utr | S5_FL |
| 19 | 3637102 | *hsa-mir-1184* | C | G | *PIP5K1C* | 3utr | S6_tFL |
| 19 | 43413365 | *hsa-mir-3157* | T | C | *PSG6* | 3utr | S1_tFL |
| 19 | 45165618 | *hsa-mir-1233* | C | T | *PVR* | 3utr | S4_FL,S4_tFL |
| 19 | 11433019 | *hsa-mir-1289* | A | C | *RAB3D* | 3utr | S2_tFL |
| 19 | 49995219 | *hsa-mir-34a* | G | T | *RPL13A* | 3utr | S1_tFL |
| 19 | 12810789 | *hsa-mir-3176* | C | G | *TNPO2* | 3utr | S1_tFL |
| 19 | 22152713 | *hsa-mir-193a* | A | T | *ZNF208* | 3utr | S2_tFL |
| 19 | 58638270 | *hsa-mir-1234* | T | G | *ZNF329* | 3utr | S3_tFL |
| 19 | 57093227 | *hsa-mir-1255a* | A | G | *ZNF470* | 3utr | S3_FL |
| 19 | 9720640 | *hsa-mir-31* | T | A | *ZNF561* | 3utr | S6_FL |
| 19 | 58291595 | *hsa-mir-203a* | A | C | *ZNF586* | 3utr | S3_tFL |
| 19 | 12256162 | *hsa-mir-4633* | T | C | *AC022415.5* | 3utr | S5_FL |
| 19 | 19257600 | *hsa-mir-1911* | G | C | *MEF2B* | coding | S1_FL,S1_tFL |
| 19 | 19260045 | *hsa-mir-1265* | T | A | *MEF2B* | coding | S1_FL,S1_tFL,S5_tFL |
| 19 | 18546106 | *hsa-mir-1301* | C | T | *ISYNA1* | coding | S4_FL,S4_tFL |
| 19 | 44738987 | *hsa-mir-3127* | G | A | *ZNF227* | coding | S1_tFL |
| 19 | 56953748 | *hsa-mir-127* | C | A | *ZNF667* | coding | S1_FL,S1_tFL |
| 20 | 55745511 | *hsa-mir-1537* | A | T | *BMP7* | 3utr | S1_FL,S1_tFL |
| 20 | 55745512 | *hsa-mir-1537* | T | A | *BMP7* | 3utr | S1_FL,S1_tFL |
| 20 | 26064296 | *hsa-mir-17* | T | G | *FAM182A* | 3utr | S1_FL |
| 20 | 26067008 | *hsa-mir-10a* | C | A | *FAM182A* | 3utr | S5_tFL |
| 20 | 50217352 | *hsa-mir-302b* | C | A | *ATP9A* | 3utr | S1_tFL |
| 20 | 55093928 | *hsa-mir-1253* | T | G | *C20orf43* | 3utr | S2_FL2,S2_FL3,S2_tFL |
| 20 | 6018638 | *hsa-mir-5007* | G | C | *CRLS1* | 3utr | S4_tFL |
| 20 | 34022953 | *hsa-mir-155* | A | T | *GDF5OS* | 3utr | S3_tFL |
| 20 | 30805120 | *hsa-mir-3920* | A | G | *POFUT1* | 3utr | S5_FL,S5_tFL |
| 20 | 33764826 | *hsa-mir-2116* | G | T | *PROCR* | 3utr | S1_FL,S1_tFL |
| 20 | 43127775 | *hsa-mir-6507* | G | A | *SERINC3* | 3utr | S5_FL |
| 20 | 45188535 | *hsa-mir-1238* | C | T | *SLC13A3* | 3utr | S1_tFL |
| 20 | 43708134 | *hsa-mir-1270* | A | C | *STK4* | 3utr | S3_tFL |
| 20 | 44004534 | *hsa-mir-1306* | C | T | *SYS1* | 3utr | S2_FL1,S2_FL2,S2_FL3,S2_tFL |
| 20 | 43120498 | *hsa-mir-3127* | G | C | *TTPAL* | 3utr | S1_tFL |
| 21 | 34169475 | *hsa-mir-3189* | G | C | *C21orf49* | 3utr | S4_tFL |
| 21 | 46021936 | *hsa-mir-1263* | A | G | *KRTAP10-7* | 3utr | S3_FL |
| 21 | 30244911 | *hsa-mir-1277* | C | T | *N6AMT1* | 3utr | S6_FL,S6_tFL |
| 22 | 36623188 | *hsa-mir-128* | C | T | *APOL2* | 3utr | S5_tFL |
| 22 | 42523897 | *hsa-mir-1972* | G | A | *CYP2D6* | 3utr | S4_tFL |
| 22 | 50529515 | *hsa-mir-103a* | A | G | *MOV10L1* | 3utr | S2_FL1 |
| 22 | 39620429 | *hsa-mir-3173* | T | A | *PDGFB* | 3utr | S3_tFL |
| 22 | 41755337 | *hsa-mir-1261* | T | A | *ZC3H7B* | 3utr | S5_tFL |
| 22 | 37425350 | *hsa-mir-1976* | G | A | *MPST* | coding | S2_FL2,S2_FL3,S2_tFL |
| X | 100518659 | *hsa-mir-3614* | A | G | *DRP2* | 3utr | S3_tFL |
| X | 100518668 | *hsa-mir-3662* | A | G | *DRP2* | 3utr | S3_tFL |
| X | 142711405 | *hsa-mir-3163* | A | C | *SLITRK4* | 3utr | S4_FL |
| X | 142712252 | *hsa-mir-31* | A | C | *SLITRK4* | 3utr | S4_FL,S4_tFL |
| X | 153009482 | *hsa-mir-147a* | C | G | *ABCD1* | 3utr | S4_FL |
| X | 69501023 | *hsa-mir-130a* | C | T | *ARR3* | 3utr | S6_tFL |
| X | 70836029 | *hsa-mir-185* | A | C | ***CXCR3*** | 3utr | S3_tFL |
| X | 68061256 | *hsa-mir-4708* | T | G | *EFNB1* | 3utr | S6_FL |
| X | 71424805 | *hsa-mir-1292* | G | A | *ERCC6L* | 3utr | S6_FL |
| X | 147030640 | *hsa-mir-495* | G | C | *FMR1* | 3utr | S4_FL,S4_tFL |
| X | 111873998 | *hsa-mir-5011* | A | C | *LHFPL1* | 3utr | S6_FL |
| X | 123511018 | *hsa-mir-1* | T | C | *ODZ1* | 3utr | S2_FL3 |
| X | 70795324 | *hsa-mir-4705* | A | G | *OGT* | 3utr | S6_FL |
| X | 153068647 | *hsa-mir-1266* | G | A | *PDZD4* | 3utr | S6_FL |
| X | 77224046 | *hsa-mir-1204* | T | TG | *PGAM4* | 3utr | S2_tFL |
| X | 37315619 | *hsa-mir-103a* | T | G | *PRRG1* | 3utr | S4_FL |
| X | 80553667 | *hsa-mir-4662a* | T | A | *SH3BGRL* | 3utr | S3_tFL |
| X | 57161584 | *hsa-mir-1291* | A | C | *SPIN2A* | 3utr | S2_FL1 |
| X | 54220266 | *hsa-mir-1245b* | A | G | *WNK3* | 3utr | S6_tFL |
| X | 21876221 | *hsa-mir-448* | A | G | *YY2* | 3utr | S4_FL |
| X | 134481773 | *hsa-mir-1305* | G | C | *ZNF449* | 3utr | S2_tFL |

**Table S2**. Recurrent mutations identified by targeted sequencing in the validation cohort and additional variants within predicted miRNA-binding sites.

| **Table 2. Gene.** | **Mutated position** | **miRNA** | **Affected FL-tFL patients (n=34)** | **Affected ntFL patients (n=21)** | **Total patients (n=55)** | **Pools healthy controls (n=5)** | | **Comments** | **UCSC Common SNPs** | | | **Location** | |
| --- | --- | --- | --- | --- | --- | --- | --- | --- | --- | --- | --- | --- | --- |
| **EZH2** | chr7:148508727 | hsa-mir-144 | 6 | 2 | 8 | | 0 | original mutation | | - | exonic | |  |
| **ARMC10** | chr7:102739179 | hsa-mir-222 | 11 | 7 | 18 | | 0 | original mutation | | - | utr_3 | |  |
| **TUBB** | chr6:30692754 | hsa-mir-1302 | 6 | 1 | 7 | | 0 | original mutation | | - | utr_3 | |  |
| **MEF2B** | chr19:19260045 | hsa-mir-1265 | 2 | 1 | 3 | | 0 | original mutation | | - | exonic | |  |
| **METTL15** | chr11:28353434 | hsa-mir-4313 | 1 | 11 | 12 | | 0 | original mutation | | - | utr_3 | |  |
| **BCL2** | chr18:60793447 | hsa-mir-5008 | 2 | 0 | 2 | | 0 | original mutation | | - | utr_3 | |  |
| **PCDH7** | chr4:30732983 | hsa-mir-329 | 2 | 2 | 4 | | 0 | original mutation | | YES | intronic | |  |
| ZNF195 | chr11:3380000 | hsa-mir-1915 | 16 | 0 | 16 | | 2 | original mutation | | YES | utr_3 | |  |
| THOC3 | chr5:175386586 | hsa-mir-371a | 2 | 13 | 15 | | 1 | original mutation | | YES | utr_3 | |  |
| TXNDC2 | chr18:9887493 | hsa-mir-2110 | 3 | 0 | 3 | | 1 | original mutation | | YES | exonic | |  |
| RC3H1 | chr1:173901940 | hsa-mir-548an | 0 | 1 | 1 | | 0 | original mutation, repetitive region | | - | utr_3 | |  |
| AQP3 | chr9:33441702 | hsa-mir-146b | 1 | 7 | 8 | | 2 | original mutation, homopolymer | | - | utr_3 | |  |
| DPY19L2 | chr12:63953768 | hsa-mir-1303 | 6 | 5 | 11 | | 4 | original mutation | | YES | utr_3 | |  |
| MYO5B | chr18:47352742 | hsa-mir-216b | 34 | 21 | 55 | | 5 | original mutation | | - | utr_3 | |  |
| MYO5B | chr18:47352754 | hsa-mir-2681 | 34 | 21 | 55 | | 5 | original mutation | | - | utr_3 | |  |
| YY2 | chrX:21876221 | hsa-mir-448 | 20 | 16 | 36 | | 5 | original mutation | | - | intronic, utr_3 | |  |
| **SERBP1** | chr1:67874057 | hsa-mir-150 | 1 | 0 | 1 | | 0 | - | | - | utr_3 | |  |
| **SH3BGRL** | chrX:80553647 | hsa-mir-4662a | 1 | 0 | 1 | | 0 | - | | - | utr_3 | |  |
| **HIST1H2AC** | chr6:26123679 | hsa-mir-3176 | 1 | 0 | 1 | | 0 | - | | - | intronic | |  |
| **SYNJ2** | chr6:158519586 | hsa-mir-2355 | 1 | 14 | 15 | | 0 | - | | - | utr_3 | |  |
| **MYO5B** | chr18:47352731 | hsa-mir-216b | 3 | 2 | 5 | | 0 | - | | YES | utr_3 | |  |
| **EZH2** | chr7:148508728 | hsa-mir-144 | 3 | 2 | 5 | | 0 | - | | - | - | |  |
| **THNSL1** | chr10:25314536 | hsa-mir-4653 | 1 | 0 | 1 | | 0 | - | | YES | utr_3 | |  |
| **SYS1-DBNDD2** | chr20:44004537 | hsa-mir-1306 | 1 | 0 | 1 | | 0 | - | | - | intronic | |  |
| **EVL** | chr14:100604644 | hsa-mir-2467 | 3 | 0 | 3 | | 0 | - | | YES | intronic | |  |
| **MEF2B** | chr19:19260055 | hsa-mir-1265 | 1 | 0 | 1 | | 0 | - | | - | exonic | |  |
| **POU1F1** | chr3:87308572 | hsa-mir-1306 | 3 | 1 | 4 | | 0 | - | | YES | intergenic | |  |
| **NCMAP** | chr1:24933795 | hsa-mir-4781 | 1 | 0 | 1 | | 0 | - | |  | utr_3 | |  |
| **SENP1** | chr12:48438315 | hsa-mir-196a | 0 | 1 | 1 | | 0 | - | | - | utr_3 | |  |
| **ATP2B4** | chr1:203709129 | hsa-mir-2682 | 1 | 0 | 1 | | 0 | - | | - | utr_3 | |  |
| **C2orf44** | chr2:24253452 | hsa-mir-302a | 1 | 0 | 1 | | 0 | - | | - | utr_3 | |  |
| **N6AMT1** | chr21:30244899 | hsa-mir-1277 | 0 | 1 | 1 | | 0 | - | | - | utr_3 | |  |
| ZNF449 | chrX:134481764 | hsa-mir-1305 | 1 | 0 | 1 | | 0 | Repetitive region | | - | intronic | |  |
| PCDH7 | chr4:30732967 | hsa-mir-329 | 0 | 1 | 1 | | 0 | Repetitive region | | - | intronic | |  |
| APOL2 | chr11:2317496 | hsa-mir-128 | 1 | 0 | 1 | | 0 | Repetitive region | | YES | utr_3 | |  |
| CD300E | chr17:72608384 | hsa-mir-3120 | 7 | 2 | 9 | | 0 | Repetitive region | | - | utr_3 | |  |
| CD300E | chr17:72608385 | hsa-mir-3120 | 1 | 0 | 1 | | 0 | Repetitive region | | - | utr_3 | |  |
| CD300E | chr17:72608386 | hsa-mir-3120 | 12 | 5 | 17 | | 0 | Repetitive region | | - | utr_3 | |  |
| CD300E | chr17:72608390 | hsa-mir-3120 | 1 | 0 | 1 | | 0 | Repetitive region | | - | utr_3 | |  |
| CD300E | chr17:72608392 | hsa-mir-3120 | 17 | 2 | 19 | | 0 | Repetitive region | | - | utr_3 | |  |
| CD300E | chr17:72608394 | hsa-mir-3120 | 16 | 5 | 21 | | 0 | Repetitive region | | - | utr_3 | |  |
| CD300E | chr17:72608396 | hsa-mir-3121 | 7 | 4 | 11 | | 0 | Repetitive region | | - | utr_3 | |  |
| CD300E | chr17:72608398 | hsa-mir-3120 | 7 | 4 | 11 | | 0 | Repetitive region | | - | utr_3 | |  |
| SIGLEC15 | chr18:43422405 | hsa-mir-342 | 2 | 6 | 8 | | 0 | Repetitive region | | - | utr_3 | |  |
| ENDOD1 | chr11:94863497 | hsa-mir-3136 | 1 | 4 | 5 | | 0 | Repetitive region | | - | utr_3 | |  |
| RAB3D | chr19:11433017 | hsa-mir-1289 | 1 | 0 | 1 | | 0 | homopolymer | | - | utr_3 | |  |
| TFAP2B | chr6:50811110 | hsa-mir-1237 | 9 | 14 | 23 | | 0 | homopolymer | | - | utr_3 | |  |
| ATP9A | chr20:50217341 | hsa-mir-302b | 0 | 3 | 3 | | 0 | homopolymer | | YES | utr_3 | |  |
| ATP9A | chr20:50217342 | hsa-mir-302b | 6 | 2 | 8 | | 0 | homopolymer | | YES | utr_3 | |  |
| ATP9A | chr20:50217343 | hsa-mir-302b | 1 | 0 | 1 | | 0 | homopolymer | | YES | utr_3 | |  |
| ATP9A | chr20:50217349 | hsa-mir-302b | 1 | 0 | 1 | | 0 | homopolymer | | - | utr_3 | |  |
| ATP9A | chr20:50217350 | hsa-mir-302b | 1 | 0 | 1 | | 0 | homopolymer | | - | utr_3 | |  |
| ATP9A | chr20:50217354 | hsa-mir-302b | 1 | 0 | 1 | | 0 | homopolymer | | - | utr_3 | |  |
| CCDC148 | chr2:159027685 | hsa-mir-1587 | 0 | 3 | 3 | | 0 | homopolymer | |  | intronic | |  |
| ENDOD1 | chr11:94863498 | hsa-mir-3136 | 17 | 1 | 18 | | 0 | homopolymer | | - | utr_3 | |  |
| AQP3 | chr9:33441701 | hsa-mir-146b | 1 | 0 | 1 | | 0 | homopolymer | | - | utr_3 | |  |
| HOXC8 | chr12:54405179 | hsa-mir-1234 | 0 | 2 | 2 | | 0 | homopolymer | | YES | utr_3 | |  |
| THNSL1 | chr10:25314538 | hsa-mir-4653 | 13 | 0 | 13 | | 1 | - | | YES | utr_3 | |  |
| SMU1 | chr9:33043718 | hsa-mir-4316 | 4 | 0 | 4 | | 1 | - | | YES | utr_3 | |  |
| SGPP2 | chr2:223423653 | hsa-mir-202 | 1 | 3 | 4 | | 1 | - | | YES | utr_3 | |  |
| OR51E2 | chr11:4702539 | hsa-mir-1292 | 1 | 1 | 2 | | 1 | Repetitive region | | YES | utr_3 | |  |
| EMP2 | chr16:10623864 | hsa-mir-4471 | 3 | 0 | 3 | | 2 | - | | - | utr_3 | |  |
| TUBB | chr6:30692768 | hsa-mir-1302 | 4 | 1 | 5 | | 2 | - | | YES | utr_3 | |  |
| PYROXD1 | chr12:21622051 | hsa-mir-103b | 2 | 4 | 6 | | 2 | - | | YES | utr_3 | |  |
| GFPT2 | chr5:179727706 | hsa-mir-3613 | 1 | 1 | 2 | | 2 | - | | YES | utr_3 | |  |
| BCL2 | chr18:60793921 | hsa-mir-27a | 2 | 1 | 3 | | 3 | - | | YES | utr_3 | |  |
| SIGLEC15 | chr18:43422409 | hsa-mir-342 | 5 | 0 | 5 | | 3 | Repetitive region | | - | utr_3 | |  |
| SIGLEC15 | chr18:43422407 | hsa-mir-342 | 16 | 10 | 26 | | 3 | Repetitive region | | - | utr_3 | |  |
| ARMC10 | chr7:102739188 | hsa-mir-222 | 6 | 5 | 11 | | 4 | - | | YES | utr_3 | |  |
| PCDH7 | chr4:30732985 | hsa-mir-329 | 5 | 4 | 9 | | 4 | - | | YES | intronic | |  |
| ATP9A | chr20:50217344 | hsa-mir-302b | 3 | 5 | 8 | | 4 | homopolymer | | YES | utr_3 | |  |
| ZNF195 | chr11:3380000 | hsa-mir-1915 | 33 | 21 | 54 | | 5 | - | | - | utr_3 | |  |
| ZNF195 | chr11:3379996 | hsa-mir-1915 | 34 | 20 | 54 | | 5 | - | | - | utr_3 | |  |
| ZNF195 | chr11:3380008 | hsa-mir-1915 | 23 | 21 | 44 | | 5 | - | | YES | utr_3 | |  |
| PLXNA4 | chr7:131812654 | hsa-mir-802 | 34 | 21 | 55 | | 5 | - | | YES | utr_3 | |  |
| IP6K1 | chr3:49763338 | hsa-mir-3675 | 29 | 18 | 47 | | 5 | - | | YES | utr_3 | |  |
| SPOCK3 | chr4:167655218 | hsa-mir-192 | 23 | 10 | 33 | | 5 | - | | YES | utr_3 | |  |
| SPOCK3 | chr4:167655235 | hsa-mir-192 | 10 | 6 | 16 | | 5 | - | | YES | utr_3 | |  |
| TXNDC2 | chr18:9887497 | hsa-mir-2110 | 30 | 21 | 51 | | 5 | - | | YES | exonic | |  |
| MYO5B | chr18:47352733 | hsa-mir-216b | 34 | 21 | 55 | | 5 | - | | - | utr_3 | |  |
| C1orf95 | chr1:226790197 | hsa-mir-30a | 14 | 7 | 21 | | 5 | - | | YES | utr_3 | |  |
| BCL2 | chr18:60793655 | miR-138-5p | 16 | 8 | 24 | | 5 | - | | YES | utr_3 | |  |
| CCDC148 | chr2:159027686 | hsa-mir-1587 | 18 | 14 | 32 | | 5 | homopolymer | | - | intronic | |  |
| CCDC148 | chr2:159027687 | hsa-mir-1587 | 1 | 0 | 1 | | 5 | homopolymer | | - | intronic | |  |
| ENDOD1 | chr11:94863499 | hsa-mir-3136 | 20 | 0 | 20 | | 5 | homopolymer | | - | utr_3 | |  |

The table shows the 16 original mutations identified in more than one sample in the validation cohort as well as the 69 additional variants identified within the predicted miRNA binding sites in this cohort. It also shows the genomic coordenates of the variants, their genomic location, the predicted targetting miRNA and the sample type were thay have been identified. The table also shows the 35 variants discarded for being present in heathy controls and the additional 27 variants excluded for being located in repetitive region or homopolymer regions. The remaining 23 variants are highlighted with bold letters.

**Table S3.** Sample detailed information.

| **Sample ID** | **Diagnosis** | **Biopsy** | **Preserved** | **Source** | **WGS cohort** | **Deep Targeted Seq cohort** |
| --- | --- | --- | --- | --- | --- | --- |
| S1FL | FL (pre-t) | LN | Frozen | BCI | Y | Y |
| S2FL1 | FL (pre-t) | LN | Frozen | BCI | Y | Y |
| S2FL2 | FL (pre-t) | LN | Frozen | BCI | Y | Y |
| S2FL3 | FL (pre-t) | LN | Frozen | BCI | Y | Y |
| S3FL | FL (pre-t) | LN | Frozen | BCI | Y | Y |
| S4FL | FL (pre-t) | LN | Frozen | BCI | Y | Y |
| S5FL | FL (pre-t) | LN | Frozen | BCI | Y | Y |
| S6FL | FL (pre-t) | LN | Frozen | BCI | Y | Y |
| S1tFL | tFL | LN | Frozen | BCI | Y | Y |
| S2tFL | tFL | LN | Frozen | BCI | Y | Y |
| S3tFL | tFL | LN | Frozen | BCI | Y | Y |
| S4tFL | tFL | LN | Frozen | BCI | Y | Y |
| S5tFL | tFL | LN | Frozen | BCI | Y | Y |
| S6tFL | tFL | LN | Frozen | BCI | Y | Y |
| 09-5129 | FL (pre-t) | LN | FFPE | CUH | N | Y |
| 09-6449-1X | tFL | LN | FFPE | CUH | N | Y |
| 09-9938A | FL (pre-t) | LN | FFPE | CUH | N | Y |
| 09-23799 | tFL | LN | FFPE | CUH | N | Y |
| 05-.2226 | FL (pre-t) | LN | FFPE | CUH | N | Y |
| 04-15557 | FL (pre-t) | LN | FFPE | CUH | N | Y |
| FL1 | FL (pre-t) | LN | Frozen | BCI | N | Y |
| FL2 | FL (pre-t) | LN | Frozen | BCI | N | Y |
| FL3 | FL (pre-t) | LN | Frozen | BCI | N | Y |
| FL4 | FL (pre-t) | LN | Frozen | BCI | N | Y |
| FL5 | FL (pre-t) | LN | Frozen | BCI | N | Y |
| FL7 | FL (pre-t) | LN | Frozen | BCI | N | Y |
| FL9 | FL (pre-t) | LN | Frozen | BCI | N | Y |
| FL10 | FL (pre-t) | LN | Frozen | BCI | N | Y |
| FL11 | FL (pre-t) | LN | Frozen | BCI | N | Y |
| FL12 | FL (pre-t) | LN | Frozen | BCI | N | Y |
| FL13 | FL (pre-t) | LN | Frozen | BCI | N | Y |
| FL14 | FL (pre-t) | LN | Frozen | BCI | N | Y |
| FL15 | FL (pre-t) | LN | Frozen | BCI | N | Y |
| FL16 | FL (pre-t) | LN | Frozen | BCI | N | Y |
| FL23 | FL (pre-t) | LN | Frozen | BCI | N | Y |
| FL25 | FL (pre-t) | LN | Frozen | BCI | N | Y |
| FL26 | FL (pre-t) | LN | Frozen | BCI | N | Y |
| FL34 | FL (pre-t) | LN | Frozen | BCI | N | Y |
| FL35 | FL (pre-t) | LN | Frozen | BCI | N | Y |
| DB1 | tFL | LN | Frozen | BCI | N | Y |
| DB2 | tFL | LN | Frozen | BCI | N | Y |
| DB3 | tFL | LN | Frozen | BCI | N | Y |
| DB4 | tFL | LN | Frozen | BCI | N | Y |
| DB5 | tFL | LN | Frozen | BCI | N | Y |
| DB7 | tFL | LN | Frozen | BCI | N | Y |
| DB9 | tFL | LN | Frozen | BCI | N | Y |
| DB10 | tFL | LN | Frozen | BCI | N | Y |
| DB11 | tFL | LN | Frozen | BCI | N | Y |
| DB12 | tFL | LN | Frozen | BCI | N | Y |
| DB13 | tFL | LN | Frozen | BCI | N | Y |
| DB14 | tFL | LN | Frozen | BCI | N | Y |
| DB15 | tFL | LN | Frozen | BCI | N | Y |
| DB16 | tFL | LN | Frozen | BCI | N | Y |
| DB23 | tFL | LN | Frozen | BCI | N | Y |
| DB25 | tFL | LN | Frozen | BCI | N | Y |
| DB26 | tFL | LN | Frozen | BCI | N | Y |
| DB34 | tFL | LN | Frozen | BCI | N | Y |
| DB35 | tFL | LN | Frozen | BCI | N | Y |
| THD15T0024PF1 | FL (pre-t) | Ovary | FFPE | DUH | N | Y |
| THD15T0025CM1 | tFL | Submaxillary gland | Frozen | DUH | N | Y |
| T120012 | ntFL | LN | Frozen | DUH | N | Y |
| T120014 | ntFL | LN | Frozen | DUH | N | Y |
| T130021 | ntFL | LN | Frozen | DUH | N | Y |
| T130023 | ntFL | LN | Frozen | DUH | N | Y |
| T130024 | ntFL | LN | Frozen | DUH | N | Y |
| T130027 | ntFL | Ileum | Frozen | DUH | N | Y |
| T130032 | ntFL | LN | Frozen | DUH | N | Y |
| THD13T0034 | ntFL | LN | Frozen | DUH | N | Y |
| THD13T0038 | ntFL | LN | Frozen | DUH | N | Y |
| THD15T0010 | ntFL | LN | Frozen | DUH | N | Y |
| THD15T0022CM1 | ntFL | LN | Frozen | DUH | N | Y |
| THD15T0023CM1 | ntFL | LN | Frozen | DUH | N | Y |
| T130070.1 | ntFL | LN | Frozen | DUH | N | Y |
| THD15T0004 | ntFL | LN | Frozen | DUH | N | Y |
| THD15T0009 | ntFL | LN | Frozen | DUH | N | Y |
| THD15T0011 | ntFL | LN | Frozen | DUH | N | Y |
| THD15T0012 | ntFL | LN | Frozen | DUH | N | Y |
| THD15T0014 | ntFL | LN | Frozen | DUH | N | Y |
| THD15T0016 | ntFL | LN | Frozen | DUH | N | Y |
| THD15T0017 | ntFL | LN | Frozen | DUH | N | Y |
| THD15T0018 | ntFL | LN | Frozen | DUH | N | Y |
| T130019 | FL (pre-t) | LN | Frozen | DUH | N | Y |
| THD13T0033 | FL (pre-t) | LN | Frozen | DUH | N | Y |
| THD13T0035 | tFL | LN | Frozen | DUH | N | Y |
| THD15T0026PF1 | tFL  (DLBCL+Burkitt) | Skin | FFPE | DUH | N | Y |

This information includes diagnosis, biopsied tissue, preservation of the sample, hospital and in what cohort the sample was included. FL = Follicular lymphoma, tFL = transformed follicular lymphoma, FL (pre-t) = pre-transformed follicular lymphoma, ntFL = non-transformed follicular lymphoma, DLBCL = Diffuse large B-cell lymphoma, LN = lymph node, FFPE = Formalin-fixed paraffin-embedded, DUH = Donostia University Hospital, CUH = Cruces University Hospital, BCI = Barts Cancer Institute.

**Supplemental Figure S1:** Distribution of the frequency of the mutations (n = 2056) identified using WGS in 6 FL cases in protein coding genes.


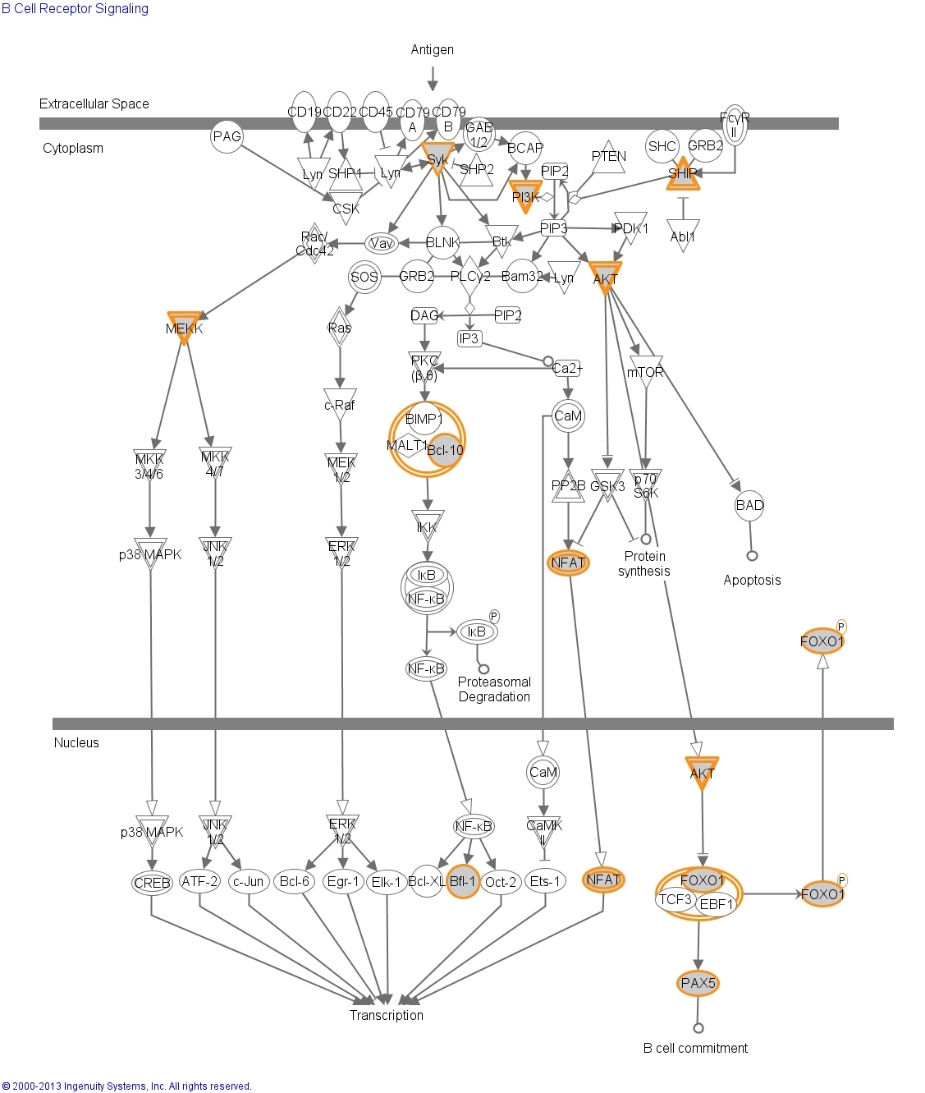


**Supplementary Figure S2:** B-cell receptor signalling pathway. Genes implicated in this signalling pathway harbouring mutations in predicted miRNA binding sites in FL patients are highlighted in yellow. These genes are SHIP, AKT, NFAT, BIMP1, FOXO1, PAX5 among others.


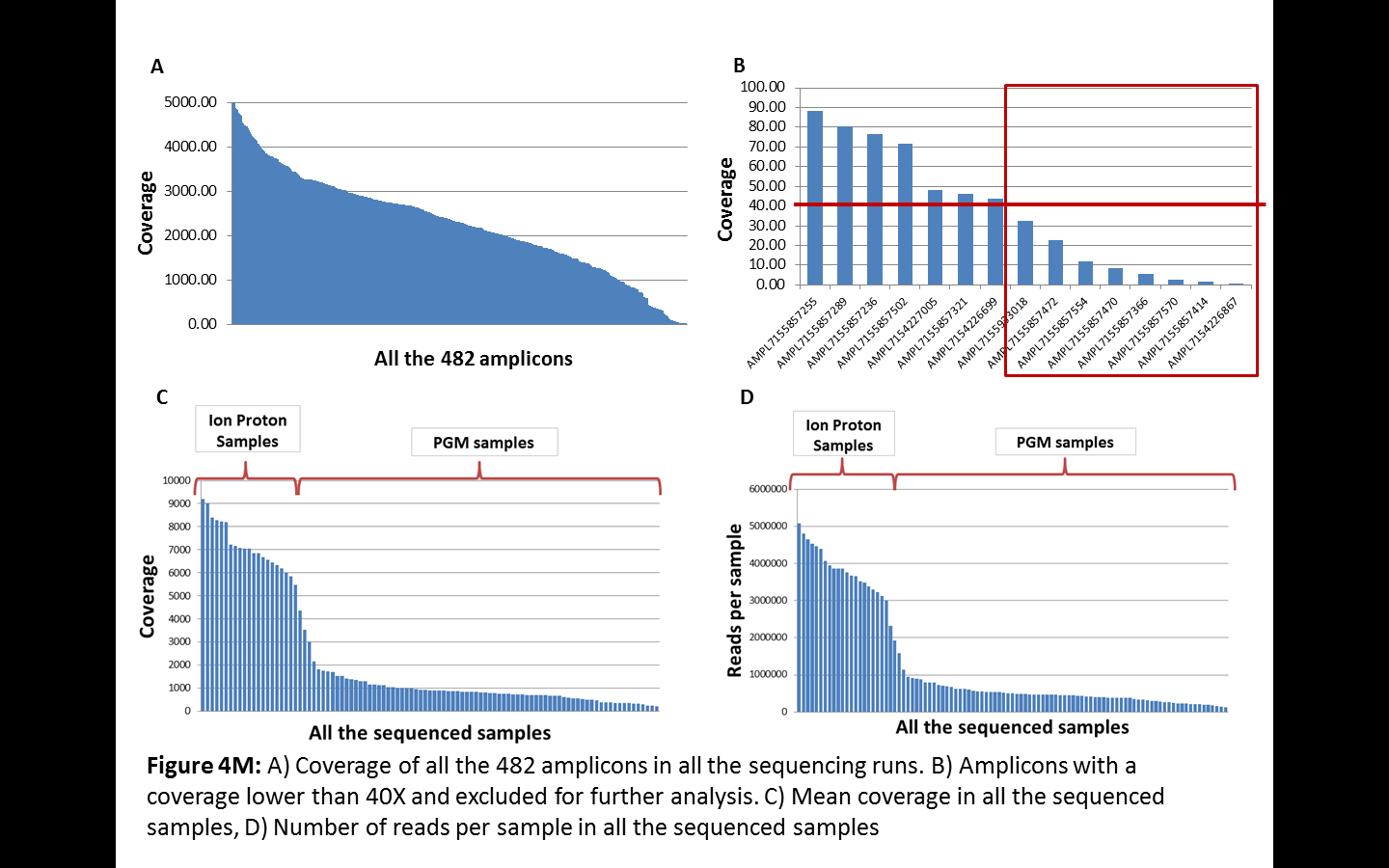


**Supplemental Figure S3****:** Sequencing coverage archived using the custom targeted sequencing panel. Ampliseq panel in PGM and Proton Ion Torrent platforms for every amplicon.


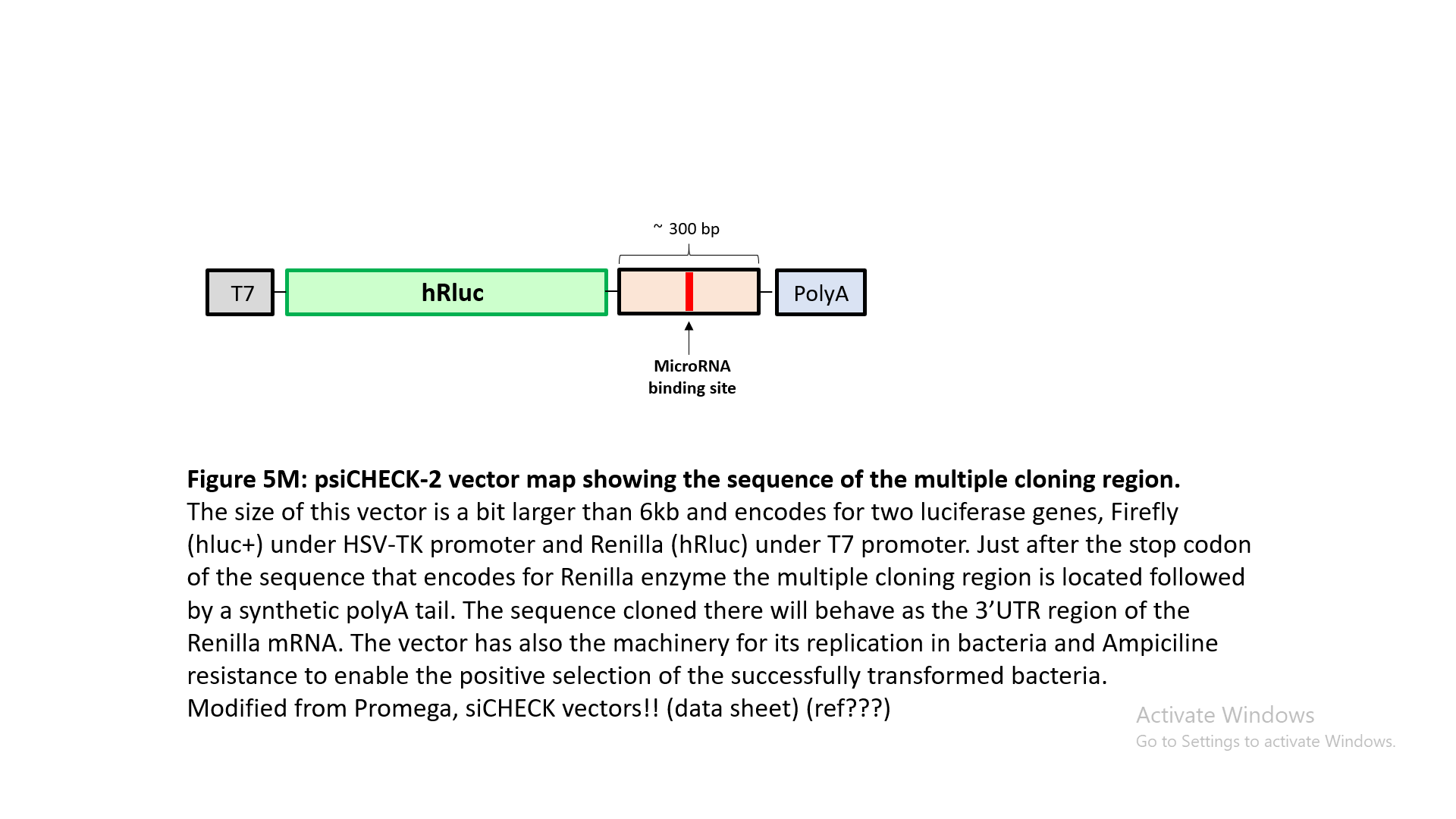


**Supplemental Figure S4:** Schematic diagram of the construct for luciferase assays in psiCHECK-2 vector. Renilla luciferase (hRluc) coding sequence is under T7 promoter. Just after the stop codon of the sequence that encodes for Renilla enzyme the multiple cloning region is located followed by a synthetic polyA tail. The sequence cloned will behave as the 3’UTR region of the Renilla. This vector also encodes for Firefly luciferase (hluc+) as a control for normalization (not shown in the figure).
